# Genome sequence of the small brown planthopper, *Laodelphax striatellus*

**DOI:** 10.1093/gigascience/gix109

**Published:** 2017-11-10

**Authors:** Junjie Zhu, Feng Jiang, Xianhui Wang, Pengcheng Yang, Yanyuan Bao, Wan Zhao, Wei Wang, Hong Lu, Qianshuo Wang, Na Cui, Jing Li, Xiaofang Chen, Lan Luo, Jinting Yu, Le Kang, Feng Cui

**Affiliations:** State Key Laboratory of Integrated Management of Pest Insects and Rodents, Institute of Zoology, Chinese Academy of Sciences, Beijing 100101, China; Beijing Institutes of Life Science, Chinese Academy of Sciences, Beijing 100101, China; State Key Laboratory of Rice Biology and Ministry of Agriculture Key Laboratory of Agricultural Entomology, Institute of Insect Sciences, Zhejiang University, Hangzhou 310058, China; University of Chinese Academy of Sciences, Beijing 100049, China

**Keywords:** comparative genomics, insects, genome sequencing, annotation, virus transmission

## Abstract

**Background:**

*Laodelphax striatellus* Fallén (Hemiptera: Delphacidae) is one of the most destructive rice pests. *L. striatellus* is different from 2 other rice planthoppers with a released genome sequence, *Sogatella furcifera* and *Nilaparvata lugens*, in many biological characteristics, such as host range, dispersal capacity, and vectoring plant viruses. Deciphering the genome of *L. striatellus* will further the understanding of the genetic basis of the biological differences among the 3 rice planthoppers.

**Findings:**

A total of 190 Gb of Illumina data and 32.4 Gb of Pacbio data were generated and used to assemble a high-quality *L. striatellus* genome sequence, which is 541 Mb in length and has a contig N50 of 118 Kb and a scaffold N50 of 1.08 Mb. Annotated repetitive elements account for 25.7% of the genome. A total of 17 736 protein-coding genes were annotated, capturing 97.6% and 98% of the BUSCO eukaryote and arthropoda genes, respectively. Compared with *N. lugens* and *S. furcifera*, *L. striatellus* has the smallest genome and the lowest gene number. Gene family expansion and transcriptomic analyses provided hints to the genomic basis of the differences in important traits such as host range, migratory habit, and plant virus transmission between *L. striatellus* and the other 2 planthoppers.

**Conclusions:**

We report a high-quality genome assembly of *L. striatellus*, which is an important genomic resource not only for the study of the biology of *L. striatellus* and its interactions with plant hosts and plant viruses, but also for comparison with other planthoppers.

## Background

**Figure 1:**
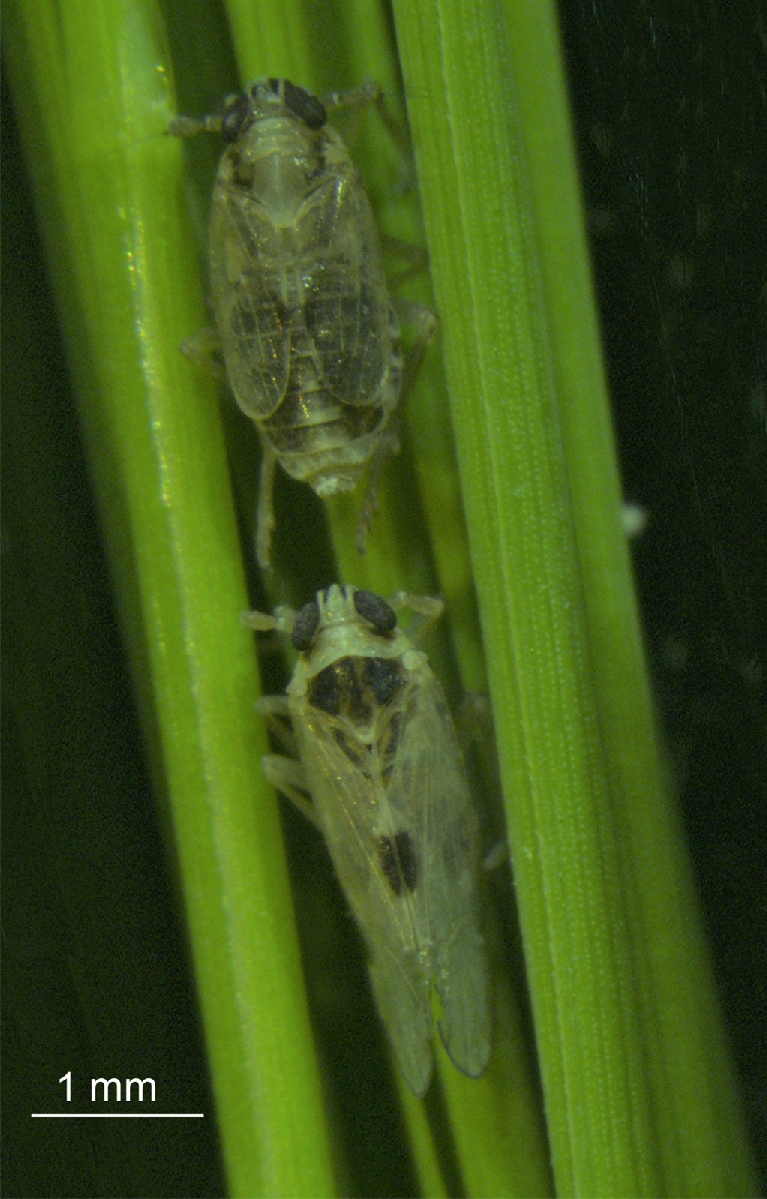
Photograph of *Laodelphax striatellus* on a rice plant leaf. Scale bar, 1 mm.

The small brown planthopper, *Laodelphax striatellus* (Delphacidae, Hemiptera), is one of the most destructive pests in a variety of crops (Fig. 1). It is widespread in the Palearctic region, including countries such as China, Japan, Germany, Italy, Russia, Kazakhstan, Turkey, and United Kingdom [1]. *L. striatellus* is polyphagous and its hosts include rice, maize, oats, tall oatgrass, wheat, and barley. It injures plants by sap-sucking behavior using its piercing-sucking mouthpart, after which symptoms like stunting, chlorosis, and hopper burn may further develop in plants. Apart from feeding damage, *L. striatellus* transmits various plant viruses, such as rice stripe virus (RSV), rice black-streaked dwarf virus (RBSDV), barley yellow striate mosaic virus, maize rough dwarf virus, wheat rosette stunt virus, and wheat chlorotic streak virus [2]. Some of these viruses may cause serious damage to agricultural production, such as RSV and RBSDV. For example, rice stripe disease caused by RSV has broken out over the past several decades in many East Asian countries, including China, where rice field production was reduced by 30–50% and total loss of harvest was observed in some areas [3].

*L. striatellus* is distinct from 2 other rice planthoppers, white-backed planthopper (*Sogatella furcifera*) and brown planthopper (*Nilaparvata lugens*), in several important traits such as host range, dispersal capacity, and plant viruses that they vector. *N. lugens* mostly feeds on rice plants, *S. furcifera* feeds on rice, wheat, and maize, and *L. striatellus* has an even broader host range. Both *N. lugens* and *S. furcifera* are known for migratory habits [4]. Whereas *S. furcifera* is the vector of Southern rice black streak dwarf virus (SRBSDV) [5] and *N. lugens* is the vector of rice ragged stunt virus (RRSV) and rice grassy stunt virus [6, 7], *L. striatellus* is the carrier of RSV, RBSDV, and several other viruses. Although the genome sequences of *S. furcifera* and *N. lugens* have been released recently [8, 9], no comparative genomic analyses were reported for the 2 planthoppers. Deciphering the genome of *L. striatellus* can help us understand the genetic basis underlying the differences in important traits between *L. striatellus* and the other 2 rice planthoppers.

## Data Description

### Sample and sequencing

The inbreeding line used for genome sequencing is an inbred laboratory strain that was derived from a field population collected in Hai’an, Jiangsu province, China. A single gravid female was selected, and her progenies were sib-mated for 22 generations to obtain the inbreeding line. Planthoppers were reared on 2–3-cm rice seedlings at 25°C and a photoperiod of 16:8 hours of light/dark. DNA was extracted by using Puregene Core Kit A (Qiagen Sciences, Maryland, USA) from the F22 specimens following the manufacturer's instruction. We built 5 libraries with insert size between 180 bp and 800 bp for paired-end sequencing and 9 libraries with insert size between 1.4 Kb and 24 Kb for mate-pair sequencing according to the standard protocols of the Illumina HiSeq 2500 sequencer (Table 1). We also constructed 33 Pacbio RSII libraries according to the standard Pacbio protocols (Table 1). In total, we generated 190 Gb of Illumina data (126 Gb of paired-end reads and 64 Gb of mated-pair reads) and 32.4 Gb of Pacbio data, representing 316× and 54× coverage of the genome, respectively.

**Table 1:** Sequencing data used for genome assembly and annotation

Category	Accession	Life stage	Sample type	Insert size, bp	Read length, bp	No. of reads
Survey	SRR5816389	Adult	DNA	230	2 × 125	127 772 669
Assmebly	SRR5830088	Adult	DNA	180	2 × 100	123 459 791
	SRR5816388	Adult	DNA	250	2 × 125	137 013 558
	SRR5816387	Adult	DNA	500	2 × 100	141 587 274
	SRR5816386	Adult	DNA	500	2 × 125	30 520 480
	SRR5816393	Adult	DNA	800	2 × 100	153 498 320
	SRR5816392	Adult	DNA	1.4–1.6 K	2 × 125	40 251 413
	SRR5816391	Adult	DNA	2.6–2.8 K	2 × 125	36 559 438
	SRR5816390	Adult	DNA	5–5.6 K	2 × 125	26 684 783
	SRR5816385	Adult	DNA	5.6–6.5 K	2 × 125	23 069 935
	SRR5816384	Adult	DNA	9–11 K	2 × 125	24 285 333
	SRR5816377	Adult	DNA	11–13 K	2 × 125	23 396 366
	SRR5816376	Adult	DNA	13–15 K	2 × 125	30 547 732
	SRR5816379	Adult	DNA	15–18 K	2 × 125	25 926 919
	SRR5816378	Adult	DNA	18–24 K	2 × 125	26 325 395
	SRR5817574	Adult	DNA	-	8559	99 701
	SRR5817559	Adult	DNA	-	8947	77 038
	SRR5817582	Adult	DNA	-	8474	104 288
	SRR5817569	Adult	DNA	-	8518	114 320
	SRR5817560	Adult	DNA	-	9202	80 599
	SRR5817562	Adult	DNA	-	9211	100 089
	SRR5817573	Adult	DNA	-	8610	102 997
	SRR5817558	Adult	DNA	-	9007	86 083
	SRR5817581	Adult	DNA	-	8452	89 374
	SRR5817570	Adult	DNA	-	8419	101 715
	SRR5817550	Adult	DNA	-	9192	82 657
	SRR5817576	Adult	DNA	-	8597	105 080
	SRR5817553	Adult	DNA	-	8586	77 467
	SRR5817557	Adult	DNA	-	8821	75 712
	SRR5817567	Adult	DNA	-	8363	106 634
	SRR5817575	Adult	DNA	-	8620	105 795
	SRR5817552	Adult	DNA	-	8985	66 096
	SRR5817556	Adult	DNA	-	8573	83 500
	SRR5817568	Adult	DNA	-	8357	104 295
	SRR5817578	Adult	DNA	-	8528	108 299
	SRR5817565	Adult	DNA	-	8728	69 694
	SRR5817555	Adult	DNA	-	8480	86 385
	SRR5817571	Adult	DNA	-	8437	106 314
	SRR5817577	Adult	DNA	-	8686	106 337
	SRR5817566	Adult	DNA	-	8890	52 889
	SRR5817554	Adult	DNA	-	8648	85 970
	SRR5817572	Adult	DNA	-	8437	101 258
	SRR5817580	Adult	DNA	-	8490	104 459
	SRR5817563	Adult	DNA	-	8954	91 218
	SRR5817561	Adult	DNA	-	8724	84 033
	SRR5817579	Adult	DNA	-	8776	107 138
	SRR5817564	Adult	DNA	-	9054	68 294
	SRR5817551	Adult	DNA	-	8508	88 776
Annotation	SRR5816381	Larva	RNA	250–300	2 × 150	23 733 333
	SRR5816380	Adult	RNA	250–300	2 × 150	24 933 333
	SRR5816383	Egg	RNA	250–300	2 × 150	24 633 333
	SRR5816382	Fat body	RNA	250–300	2 × 150	31 300 000
	SRR5816375	Brain	RNA	250–300	2 × 150	40 333 333
	SRR5816374	Gonad	RNA	250–300	2 × 150	33 300 000
	SRR5816394	Tentacle	RNA	250–300	2 × 150	24 966 666

Survey library in the Category column was used to estimate the genome size of *Laodelphax striatellus*. Libraries of insert size >1 Kb were mate-paired. For gene annotation, data from 2 previously sequenced tissues were used under accession SRR1619428 for salivary gland and SRR1617617 for alimentary canal.

For transcriptome sequencing, total RNA was isolated from 4 tissues (antenna, brain, fatty body, and gonad) and whole bodies of 3 developmental stages (egg, nymph, adult) of *L. striatellus* using TRIzol reagent (Invitrogen, Carlsbad, CA, USA) according to the manufacturer's protocol. Nanodrop (Thermo Scientific, Wilmington, DE, USA) was used to determine RNA quantity, and gel electrophoresis was used to examine RNA quality. cDNA libraries were constructed according to the manufacturer's instructions and sequenced on an Illumina HiSeq 2500 sequencer.

### Estimation of genome size and determination of chromosome number

We estimated the genome size of *L. striatellus* using 2 independent approaches: flow cytometry [10] and *k*-mer analyses [11]. The flow cytometry analysis was carried out according to a published procedure [10]. Briefly, a female adult was ground in PBS-T buffer. The mixture was filtered by a 40-μm cell filter, incubated with 2 μg/mL RNase A at 37°C for 15 minutes, and then stained with 5 μg/mL propidium iodide at 25°C for 30 minutes. The fluorescence signal was detected by a FACSCallbur Analyzer (Becton-Dickinson, San Jose, CA, USA). Heads of *Drosophila melanogaster* and cytoblasts of *Gallus gallus* were treated with the same procedure as genome size references. The genome sizes of *D. melanogaster* and *G. gallus* are known to be 0.18 pg and 1.25 pg, respectively [12]. As shown in Fig. S1, the genome size of *L. striatellus* was estimated to be 0.60 pg (587 Mb) by the flow cytometry method. In *k*-mer analysis, 31.94 Gb of clean reads were utilized to generate a *k*-mer (k = 17) depth distribution curve (Fig. S1D), based on which the genome size was estimated to be 550 Mb. Accordingly, the haploid genome size of *L. striatellus* was estimated to be 550–587 Mb.

The chromosome number was determined by cytological analysis of testes cells. The testes of newly emerged males were dissected in insect Ringer solution, fixed in Carnoy's fixative for 15 minutes. The testes were washed with 0.01 mol/L PBS solution, stained at 0.5 μg/mL Hoechst 33 258, and sealed with Antifade Mounting Medium (Beyotime, Jiangsu, China). Cells in meiosis phase were selected for chromosome counting under a confocal microscope Zeiss LSM710 (Carl Zeiss, Oberkochen, Germany). In most cases, 15 haploid chromosomes were observed (30 for diploid chromosomes) (Fig. S2), although sometimes only 14 were visible. Thus the number of chromosomes in *L. striatellus* was determined to be 2n = 30.

### Genome assembly and assessment

We assembled the genome with both Illumina sequencing and Pacbio sequencing data. Illumina data were used to build contigs and scaffolds as follows. First, all reads with ≥10% unidentified nucleotides, or with >10 nt aligned to the adapter sequences, or being putative PCR duplicates were removed to obtain clean reads. Mate-pair reads from libraries with insert sizes >2 kb were classified as paired-end, unpaired, negative, and mate-pair reads, and only the negative and mate-pair reads were retained for the assembly. Second, we employed SOAPdenovo v. 3.0 (SOAPdenovo, RRID:SCR_010752) [13,14] with the parameters “pregraph -K 33 -p 30 -d 30; contig –k 33 –M 3” to build de Bruijn graph and assemble sequencing reads into contigs. Third, all mate-pair reads were mapped to the contigs, and mate-pair information was added in a stepwise manner to connect contigs into scaffolds. GapCloser v. 1.12 (GapCloser, RRID:SCR_015026) [13] was used to fill the gaps between scaffolds with a local assembly strategy. Afterwards, PBJelly v. 15.8.24 (PBJelly, RRID:SCR_012091) [15] was used to fill the gaps between scaffolds using the 32.4 Gb (∼54×) of Pacbio data. Briefly, all the gaps (length > 25 bp) on the assembly were identified first, and the Pacbio reads were mapped to the assembly using PBJelly. The BLASR alignments were parsed to identify gap-supporting reads by comparing aligned and unaligned base positions within each read [16]. Overlap-layout-consensus engine ALLORA within PBSuite (v. 15.8.24, Pacific Biosciences Menlo Park) [17] was used to assemble the reads for each gap to generate consensus gap-filling sequences. As the final step, the consensus gap-filling sequences were spliced into the corresponding gap position in the draft assembly, replacing all Ns if the gap was closed and leaving the appropriate number of Ns if the gap was only reduced.

With the above assembly procedure, we obtained a final assembly of 541 Mb, having 38 193 scaffolds with a contig N50 length of 118 Kb and a scaffold N50 length of 1.1 Mb. The length of the assembly accounts for 91.7% and 98.4% of the estimated genome size by flow cytometry and *k*-mer analysis, respectively. The longest contig and scaffold were 2.0 Mb and 10.4 Mb, respectively (Table 2). The Pacbio sequencing data greatly improved the length of contigs compared with the published genomes of *N. lugens* (contig N50, 24.2 Kb [8]) and *S. furcifera* (contig N50, 70.7 Kb [9]), which were assembled with Illumina data only (Table 2). We aligned clean reads onto the genome assembly using BWA (BWA, RRID:SCR_010910) [18] and calculated the fraction of bases at a given sequencing depth. The results showed a very small fraction of low-coverage bases, suggesting high coverage and accuracy of the genome assembly (Fig. S3).

**Table 2:** Statistics comparison of genome assembly and annotation among 3 planthoppers

	*Laodelphax striatellus*		*Nilaparvata lugens* ^a^		*Sogatella furcifera* ^b^	
Category	Contig	Scaffold	Contig	Scaffold	Contig	Scaffold
Total size, Mb	530.2	541.0	993.8	1140.8	673.9	720.7
Total number	48 574	38 193	80 046	46 558	50 020	20 450
Maximum length, Kb	1990	10 350	230	2254	800	12 789
N50 length, Kb	118	1085	24	357	71	1185
GC content, %		34.5		34.6		31.6
TE proportion, %		23.0		38.9		39.7
BUSCO evaluation, %		92		81		92
Gene number		17 736		27 571		21 254
Average gene length, bp		14 342		11 216		12 597
Average CDS length, bp		1289		1135		1526
Average exon per gene		6		4		6
Average exon length, bp		213		264		240
Average intron length, bp		2587		3062		2064

Gene number means number of protein-coding genes.

BUSCO: benchmarking universal single copy ortholog; CDS: coding sequence; TE: transposable element.

a From the published *Nilaparvata lugens* genome [8].

b From the published *Sogatella furcifera* genome [9].

### Validation and quality control

The completeness and accuracy of the genome assembly were assessed by 4 independent approaches. First, the overall base composition and the percentage of Ns were calculated. As shown in Table S1, the assembled genome had a low percentage (1.99%) of Ns and an expected base composition, which is similar to that of the other 2 planthoppers. The overall GC content of *L. striatellus* was 34.54%, similar to that of *N. lugens* [8] and slightly higher than that of *S. furcifera* [9]. Second, we remapped Illumina paired-end reads to the assembly using BWA [18], and we found that 93.2% of reads could be mapped back, covering 96.83% of the assembled genome, including 95.08% of the genome with ≥×20 coverage (Table S2). Third, we performed *de novo* transcriptome assembly using Trinity v. 2.0.2 (Trinity, RRID:SCR_013048) for RNA-seq data from multiple developmental stages and tissues (Table 1). We also included 2 published RNA sequencing datasets from salivary glands and alimentary canal [19] in the transcriptome assembly. We mapped the assembled transcripts to the genome assembly using TopHat (TopHat, RRID:SCR_013035) with default parameters and found that 90.31% of the transcripts with >90% transcript coverage were aligned to 1 scaffold (Table S3), indicating that most expressed genes were correctly assembled in the genome. When the RNA reads from the 9 transcriptome datasets were directly mapped to the genome, 78% to 94% could be correctly mapped to the genome with appropriate splicing, indicating that the genome assembly had a good representative of gene regions (Table S4). Finally, the benchmarking universal single-copy orthologs v. 1 (BUSCO, RRID:SCR_015008) dataset representing 2675 genes for arthropoda was used for genome assessment [20]. Our assembled genome captured 92% (2470/2675) of the BUSCO genes, suggesting that a gene repertoire was nearly complete (Table S5). Taken together, these results suggest that our assembled genome was highly accurate and nearly covered the whole genome.

### Annotation of repetitive elements

Two independent methods, namely homology-based and *de novo* prediction, were applied for repetitive element annotation. For the homology-based method, the assembled genome was compared with Repbase, issued on 13 January 2014 [21], using RepeatMasker v. 4.0.5 (RepeatMasker, RRID:SCR_012954) and RepeatProteinMasker (v. 1.36) with default settings [22]. For the *de novo* prediction, we built a *de novo* repeat library with LTR_FINDER v. 1.0.5 (LTR_Finder, RRID:SCR_015247) [23], Piler (v. 1.06) [24], RepeatScout v. 1.0.5 (RepeatScout, RRID:SCR_014653) [25], and RepeatModeler v. 1.0.8 (RepeatModeler, RRID:SCR_015027). Tandem Repeat Finder (v. 4.07b) [26] was used to search tandem repeats. Furthermore, RepeatProteinMask [22] was used to identify putative transposable element (TE)–related proteins. After merging all the repetitive elements identified by abovementioned tools, we identified a total of 139.1 Mb of repetitive sequences, accounting for 25.7% of the genome (Table S6). The percentage of repetitive elements in the *L. striatellus* genome was much lower than those of *N. lugens* (48.6%) [8] and *S. furcifera* (44.3%) [9]. Of all the repetitive sequences, 10.59% were the class I transposable elements (retrotransposon), including 5.01% long interspersed nuclear elements, 1.32% long terminal repeats, and 4.26% short interspersed nuclear elements. Class II elements (DNA transposons) represented only 4.92% of the genome (Table 3). *L. striatellus* had the lowest TE fraction and the smallest genome size compared with *N. lugens* and *S. furcifera* (Table 3).

**Table 3:** Comparison of transposable element contents of the 3 planthoppers

	*Laodelphax striatellus*						*Nilaparvata lugens*		*Sogatella furcifera*	
	*De novo* + Repbase		TE proteins		Combined TEs		Combined TEs		Combined TEs	
Class	Length, bp	% of genome	Length, bp	% of genome	Length, bp	% of genome	Length, bp	% of genome	Length, bp	% of genome
DNA	24 818 676	4.59	2 550 902	0.47	26 592 872	4.92	162 024 958	14.20	126 002 323	17.33
LINE	24 160 245	4.47	4 889 094	0.90	27 124 925	5.01	182 652 892	16.00	69 257 982	9.52
LTR	7 122 249	1.32	0	0.00	7 122 249	1.32	168 492 299	14.80	31 286 552	4.30
SINE	22 739 683	4.20	743 909	0.14	23 044 510	4.26	8 272 412	0.70	10 730 722	1.48
Other	0	0.00	0	0.00	0	0.00	41 262	0.00	23 167 338	3.18
Unknown	27 609 625	5.10	0	0.00	27 609 625	5.10	21 890 733	1.90	28 395 639	3.90
Total	119 645 576	22.12	8 177 428	1.51	124 360 921	22.99	443 765 874	38.90	288 840 556	39.73

*De novo* + Repbase refers to TE integrated between *de novo* and Repbase prediction. TE proteins refers to TE identified by RepeatProteinMask. Combined TEs refers to the 2 TE combined results above. Other means TE that can be classified but doesn’t belong given classes. Unknown means TE that can’t be classified.

DNA: DNA transposon; LINE: long interspersed nuclear element; LTR: long terminal repeat; SINE: short interspersed nuclear element.

### Annotation of protein-coding genes

**Figure 2:**
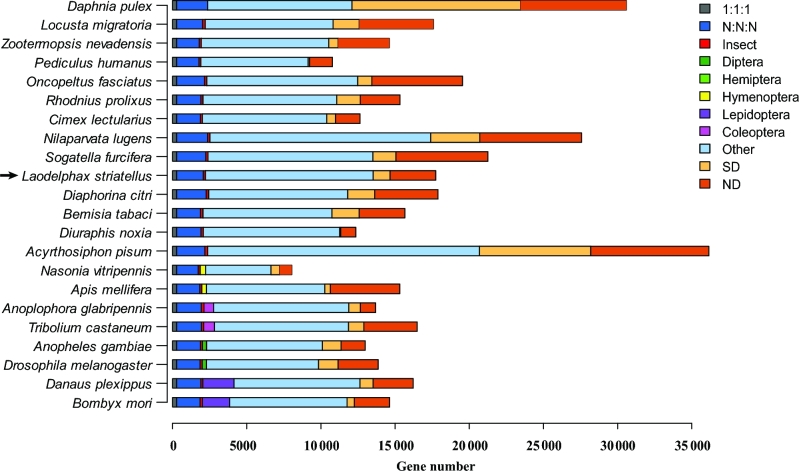
Gene cluster analysis among 22 arthropod species. 1:1:1 and N: N: N represent universal orthologs with single-copy or multiple-copy numbers, respectively. Insect, Diptera, Hemiptera, Hymenoptera, Lepidoptera, and Coleoptera stand for taxon-specific orthologs, respectively. Other indicates orthlogs that do not belong to any abovementioned ortholog categories. SD indicates species-specifically duplicated genes. ND indicates genes that cannot be classified into any other categories. The location of *Laodelphax striatellus* is indicated by an arrow.

The protein-coding genes were annotated with evidence from the homology-base method, *ab initio* prediction, and RNA-seq data. For the homology-based method, the annotated gene sets from 8 species, *N. lugens*, *Acyrthosiphon pisum*, *Pediculus humanus*, *Nasonia vitripennis*, *D. melanogaster*, *Bombyx mori*, *Rhodnius prolixus*, and *Daphnia pulex* (Table S7), were aligned to the *L. striatellus* genome using TBLASTN (TBLASTN, RRID:SCR_011822) [27] with an E-value cutoff of 1E^−5^. GeneWise v. 2.2.0 (GeneWise, RRID:SCR_015054) [28] was used to define gene models. For *ab initio* prediction, we utilized Augustus v. 3.1 (Augustus: Gene Prediction, RRID:SCR_008417) [29], GlimmerHMM v. 3.0.4 (GlimmerHMM, RRID:SCR_002654 [30], SNAP (v. 2013–11-29) [31], GeneID (v. 1.4) [32, 33], and GENSCAN v. 1.0 (GENSCAN, RRID:SCR_012902) [34] to predict potential protein-coding genes from the repeat-masked genome. Furthermore, we identified gene structures with the assistance of 9 transcriptomes assembled by Tophat-Cufflinks (v. 2.2.1) [35] and Trinity-PASA (v. 2.0.2) [36], respectively. Then we integrated all predicted gene structures above with EvidenceModeler (v. 1.1.1) [37] to obtain a nonredundant set of 17 736 protein-coding genes with an average gene length of around 16.17 Kb (Table S8–S9, Fig. S4). We constructed the orthologous gene families using annotated genes from 22 closely related species (Table S7) and found that *L. striatellus* had 4210 species-specific genes, fewer than those of *N. lugens* (10 163) and *S. furcifera* (7743) (Fig. 2). This may be attributed to the smaller genome size and lower gene number in *L. striatellus*.

We used 3 methods to evaluate the gene models that we obtained. First, we examined the 2 Kb upstream and downstream regions of annotated genes and found that the majority (16 525, 93.17%) of genes did not contain any ambiguous bases (Ns) in the 2 Kb up- and downstream regions, indicating that these gene models are not located near an assembly gap and thus the gene models are unlikely to be a fragment. Second, we compared our annotated genes with the corresponding orthlogous genes in *D. melanogaster*. We performed BLASTX (BLASTX, RRID:SCR_001653) [27] searches against the *D. melanogaster* gene set using the *de novo* assembled transcripts in *L. striatellus*. A total of 8484 assembled transcripts that had identity >60% with a *D. melanogaster* gene and covered >90% of the coding region were regarded as full-length transcripts. Among them, 3728 transcripts (excluding redundant protein isoforms) containing a complete ORF were searched against the annotated genes, and 3093 (82.97%) of them had a near perfect match to an annotated gene, indicating that most annotated genes were complete. Third, we compared our annotated genes to the 2 sets of BUSCO (v. 2) genes (1066 arthropoda genes and 303 eukaryote genes) [20] and found that our predicted genes were considered complete BUSCO genes in 97.6% and 98.0% of the eukaryote genes and arthropoda genes, respectively (Fig. S5), suggesting that a nearly complete repertoire of protein-coding gene set was determined.

To estimate the level of heterozygosity in the gene model, we aligned 23× reads to the genome assembly with BWA [18]. After removing duplicates, heterozygous SNPs were identified using BCFtools [38]. The heterozygous SNPs in the coding regions of each gene were used to compute read coverage and heterozygosity. Only a single heterozygosity peak of around 0.3 was detected (Fig. S6A). We ranked the heterozygosity rate of all the gene set and took the top 20% as high heterozygosity (the remainder was designated low heterozygosity). Coverage histograms of high and low heterozygosity showed similar ranges of coverage distribution (Fig. S6B). Therefore, the heterozygosity did not influence the gene annotation.

**Figure 3:**
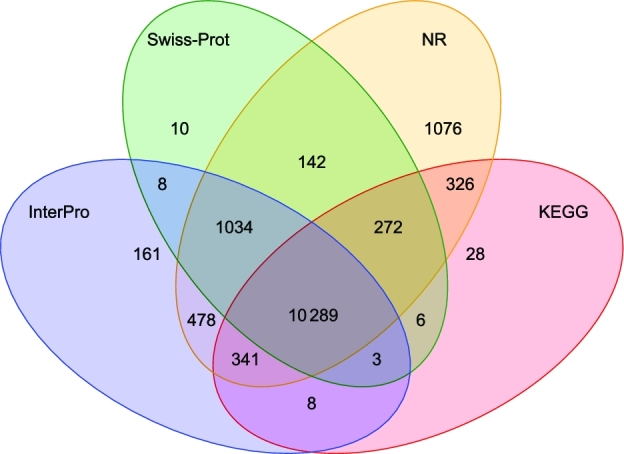
Venn diagram of functional annotation by 4 databases. NR: nonredundant protein databases.

In order to obtain putative functional assignments to the annotated genes, we compared the annotated protein sequences of *L. striatellus* to proteins in the Kyoto Encyclopedia of Genes and Genomes (KEGG, RRID:SCR_012773) [39], NR [40], and Swiss-Prot [41] databases using BLASTP (BLASTP, RRID:SCR_001010) [27] with an E-value cutoff of 1E^−5^. Domains and motifs were scanned in Interpro [42] database by InterProScan (InterProScan, RRID:SCR_005829) [43]. There were 78.7%, 66.3%, 63.6%, and 69.5% of annotated proteins showing significant sequence similarity with the proteins in NR, Swiss-prot, KEGG, and InterPro (InterPro, RRID:SCR_006695), respectively. Among the 12 322 genes with an InterPro hit, 11 159 (90.6%) had Pfam (Pfam, RRID:SCR_004726) annotations and 8935 (72.5%) had gene ontology (GO, RRID:SCR_002811) associations. After removing redundancy, 14 182 of 17 736 genes (80.0%) were assigned to known databases (Fig. 3). Among the 3554 unannotated genes, 1391 (7.8%) were *L. striatellus*–specific genes.

### Gene orthology prediction

**Figure 4:**
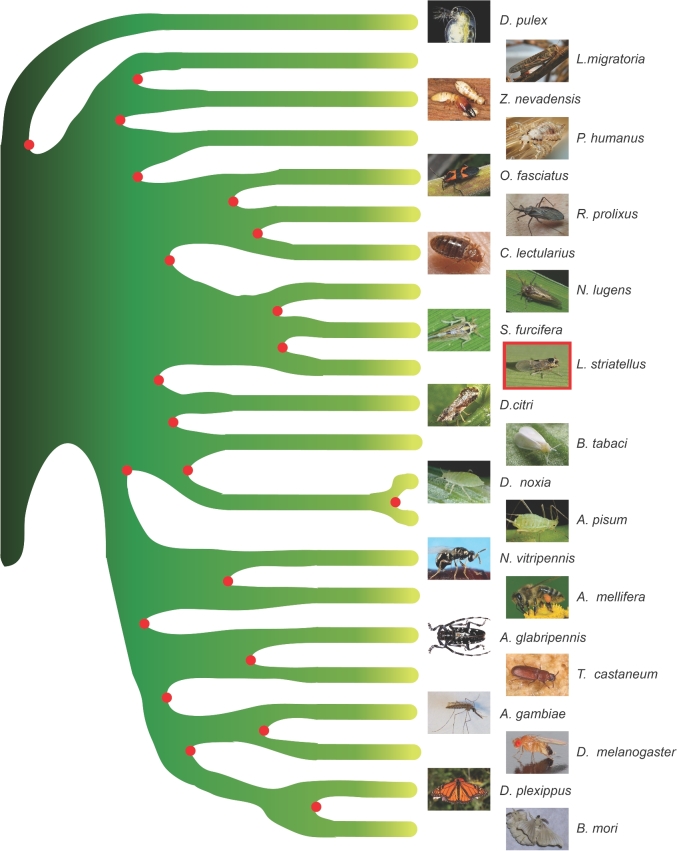
Phylogenetic analysis of 22 arthropod species. The phylogenetic tree was constructed based on amino acid sequences of 277 single-copy orthologs among 22 arthropod species (*Anopheles gambiae*, *Anoplophora glabripennis*, *Apis mellifera*, *Acyrthosiphon pisum*, *Bombyx mori*, *Bemisia tabaci*, *Cimex lectularius*, *Diaphorina citri*, *Drosophila melanogaster*, *Diuraphis noxia*, *Danaus plexippus*, *Daphnia pulex*, *Locusta migratoria*, *Laodelphax striatellus*, *Nilaparvata lugens*, *Nasonia vitripennis*, *Oncopeltus fasciatus*, *Pediculus humanus*, *Rhodnius prolixus*, *Sogatella furcifera*, *Tribolium castaneum*, *Zootermopsis nevadensis*) using the maximum likelihood algorithm. The tree was rooted with *D. pulex*.

Twenty-one sequenced insects (*Zootermopsis nevadensis*, *Tribolium castaneum*, *Anoplophora glabripennis*, *Anopheles gambiae*, *D. melanogaster*, *A. pisum*, *Diuraphis noxia*, *Cimex lectularius*, *L. striatellus*, *R. prolixus*, *N. lugens*, *S. furcifera*, *Diaphorina citri*, *Oncopeltus fasciatus*, *Apis mellifera*, *N. vitripennis*, *B. mori*, *B. tabaci*, *Danaus plexippus*, *Locusta migratoria*, and *P. humanus*) and 1 noninsect arthropoda sequenced species (*D. pulex*) were used to infer gene orthology and reconstruct the phylogenetic tree. The annotated coding sequences were downloaded from the websites listed in Table S7. The homologous gene families were identified using TreeFam [44, 45] and ascribed in different categories (Fig. 2). The gene families were identified following these steps: (i) BLASTP [27] was used to compare all protein sequences for the 22 species with an E-value cutoff of 1E^−7^; (ii) the blast alignments were concatenated by Solar (v. 0.9.6) [45], followed by homology identification among protein sequences; and (iii) gene families were identified using hcluster_sg (v. 0.5.0) [45]. RAxML (v. 8.0.19) [46] was used to reconstruct the phylogenetic tree based on the concatenated single-copy protein sequences under the PROTGAMMAAUTO model with 100 bootstrap replicates. R8s (v. 1.7.1) [47] and MCMCtree (PAML package, v. 4.7; PAML, RRID:SCR_014932) [48] were used to estimate the divergence times among species. The parameters used in MCMCtree were “–rootage 510 -clock 3 -alpha 0.977999 -model 7.” To examine gene family expansion and contraction in the 3 planthoppers, we chose 1 additional hemipteran species, *R. prolixus*, as outgroup to infer expanded/contracted gene families using CAFE (v. 3.1) [49]. A conditional *P*-value was calculated for each gene family, and the gene families with *P*-values <0.05 were considered as significantly expanded or contracted. The phylogenetic analysis revealed that *L. striatellus* clustered together with the other 2 planthoppers and had a closer relationship to *S. furcifera* than *N. lugens* (Fig. 4). The divergence times of nonplanthopper insect species were generally consistent with those estimated in the previous study [8]. The results of molecular dating analysis indicated that the ancestor of *L. striatellus* and *S. furcifera* split with *N. lugens* about 87.5 million years ago and that *L. striatellus* diverged from *S. furcifera* approximately 31 million years ago (Fig. S7).

Compared with *N. lugens* and *S. furcifera*, *L. striatellus* had fewer expanded gene families and more contracted gene families (Fig. S8). This might partially explain why *L. striatellus* has the lowest gene number among the 3 planthopper species. Since the divergence of *L. striatellus* and *S. furcifera*, *L. striatellus* and *S. furcifera* have had 95 and 547 expanded gene families, respectively (Fig. S8). The significantly expanded gene families in *L. striatellus* included some specific members of multigene families, such as odorant receptor, cytochrome P450, and serine protease (especially trypsin) (Table S10). The specific members of chemosensory protein, odorant binding protein, carboxylesterase, and ATP-binding cassette transporter families were also increased in *L. striatellus* although their *P*-values were higher than 0.05 (Table S10). Expansion of these gene families may have contributed to the widest host plant range of *L. striatellus* among the 3 planthoppers. The specific members of gene families associated with energy metabolism were significantly expanded in *S. furcifera*, such as acyl-CoA synthetase, fatty acyl-CoA reductase, acyl-CoA-binding protein, and acyl-coenzyme A thioesterase. The specific members of glyceraldehyde-3-phosphate dehydrogenase, D-beta-hydroxybutyrate dehydrogenase, ADP/ATP translocase, acyl-CoA transporter, and ATP synthase families also increased, although with *P*-values higher than 0.05 (Table S10). *N. lugens* had 433 expanded gene families (Fig. S8). A bunch of specific members from energy metabolism–related gene families, including Delta(3,5)-Delta(2,4)-dienoyl-CoA isomerase, ATP-citrate synthase, malonyl-CoA decarboxylase, NADH dehydrogenase (ubiquinone) 1α subcomplex subunit 7 and subunit 8, acyl-CoA synthetase, ATP synthase, and enoyl-CoA delta isomerase increased in *N. lugens* although their *P*-values were higher than 0.05 (Table S10). Expansion in the energy metabolism–related gene families is in accordance with the migratory habits of *S. furcifera* and *N. lugens*.

### Olfaction and detoxification system

It is essential for herbivorous insects to recognize and locate their host plants utilizing their sense of gustation and olfaction. Chemicals from the environment are received and recognized by chemoreceptor genes, including odorant receptors (ORs), gustatory receptors (GRs), and ionotropic receptors (IRs) in gustatory and olfactory organs. Detoxification gene families also play an essential role in defense against natural xenobiotics from host plants or synthetic xenobiotics including insecticides. To identify chemoreception and detoxification-related genes in *L. striatellus*, we retrieved corresponding gene sequences of other insect species from previous studies and used them as queries. These genes were searched against the *L. striatellus* gene set using BLASTP [27] with an E-value cutoff of 1E^−5^. In addition, we scanned the gene sets of 3 planthoppers for domain information using InterProScan and extracted genes with domains corresponding to each family. Finally, we integrated results from both BLASTP and InterProScan to obtain the final set of protein families.

There were 106 ORs, 38 IRs, and 12 GRs identified in *L. striatellus* (Table S11). The numbers of ORs and GRs in *L. striatellus* were more than twice as many as those in *N. lugens* and *S. furcifera*, representing a significant expansion in these 2 families. This is consistent with the fact that *L. striatellus* is the most polyphagous among the 3 planthoppers because polyphagous insects tend to have more OR genes than monophagous [8]. Moreover, we identified 2 protein families important for odor recognition and pheromone perception, namely odorant binding proteins (OBPs) and chemosensory proteins (CSPs). There were 16 OBPs and 31 CSPs in *L. striatellus*, the most among the 3 planthoppers (Table S11). The relatively higher number of odor-related genes in *L. striatellus* might be closely related to its polyphagous habit.

We manually annotated families of detoxification-related genes, including 26 UDP-glycosyltransferases, 29 glutathione-S-transferases, 54 carboxyl/cholinesterase, 73 ATP-binding cassette transporters, and 76 cytochrome P450s in *L. striatellus* (Table S12). The total number of detoxification-related genes in *L. striatellus* was smaller than that in *N. lugens*, but larger than that in *S. furcifera*.

### Immune-related genes

We identified immune gene repertoires of the 3 planthoppers using a homology-based method. Immune genes from *D. melanogaster*, *A. gambiae*, *Aedes aegypti*, and *Culex quinquefasciatus* were downloaded from ImmunoDB [50]. Gene sets from the 3 planthoppers were used as queries and searched against the immune genes of the 4 insects, respectively, using BLASTX with an E-value cutoff of 1E^−5^. The best hits were selected for further domain architecture analysis using InterProScan and then were confirmed manually. The number of immune-related genes in *L. striatellus* was 330, which was more than that in *N. lugens* (289) and *S. furcifera* (280) (Table S13). The redundant copies of immune genes in *L. striatellus* mainly included autophagy genes, 1,3-beta-D glucan binding protein genes, clip-domain serine protease genes, and genes of small RNA regulatory pathway members. However, the numbers of C-type lectin genes and Toll-like receptor genes were lower in *L. striatellus* compared with the other 2 planthoppers.

### Transcriptomic responses of 3 planthoppers to their borne plant viruses

*L. striatellus*, *S. furcifera*, and *N. lugens* transmit different rice viruses. To explore the molecular response to respective plant viruses, we analyzed and compared the transcriptomic responses of *L. striatellus* to RSV, *S. furcifera* to SRBSDV, and *N. lugens* to RRSV. The 3 viruses are transmitted in a persistent-propagative way. For *L. striatellus*, RSV was incubated in the fourth-instar nymphs for 5 days, as described previously [51]. Three replicates of infected or noninfected insects were used to construct paired-end RNA-seq libraries for sequencing on an Illumina Hiseq 2500 sequencer. The transcriptomic data of *S. furcifera* infected with SRBSDV were retrieved from a previous study [52]. The third-instar nymphs of *N. lugens* were infected by RRSV for 7 days before being collected for RNA extraction using the SV Total RNA Isolation System (Promega, Madison, WI, USA). The gene expression libraries for RRSV-infected and noninfected samples were constructed and sequenced on an Illumina HiSeq 2000 sequencer. RNA-seq reads were mapped to the corresponding genome using TopHat2 (v. 2.1.1) [53]. For *L. striatellus* and *S. furcifera*, HTSeq [54] was used to count the number of reads mapped to each gene model, and the edgeR package was used to identify differentially expressed genes (DEGs) with a fold change cutoff of 2 and FDR cutoff of 0.01. For *N. lugens*, generalized fold change for ranking differentially expressed genes from RNA-seq data was used to detect DEGs without biological replicates. The gene annotation files were downloaded from the corresponding websites (Table S14). We referred to genes with higher expressions in the viruliferous group as upregulated genes and lower as downregulated. The results showed that 460 (185 up and 275 down), 162 (48 up and 114 down), and 1070 (515 up and 555 down) genes were differentially expressed in *L. striatellus*, *N. lugens*, and *S. furcifera*, respectively, when bearing their respective plant viruses.

The DEGs in the 3 planthoppers were compared in GO terms, and the common GO terms were retrieved (Table S15). The upregulated genes in the 3 planthoppers were involved in the biological processes of regulation of transcription (GO:0 006355) and protein phosphorylation (GO:0 006468). The downregulated genes in the 3 planthoppers took part in the biological processes of carbohydrate metabolic process (GO:0 005975), chitin catabolic process (GO:0 006032), and proteolysis (GO:0 006508).

Two zinc finger proteins of *L. striatellus*, 1 zinc finger protein of *N. lugens*, and 6 zinc finger proteins of *S. furcifera* were commonly upregulated while genes of chitinases, cytochrome P450 CYP4s, and trypsins were commonly downregulated in the 3 planthoppers (Table S16) in response to their respective plant viruses. We also identified homologous genes that were commonly regulated in the 3 planthoppers by aligning *N. lugens* DEGs with those of *L. striatellus* and *S. furcifera* using BLASTP with a cutoff of 1E^−3^, a sequence identity higher than 60%, and a coverage higher than 50%. Three groups of homologous genes, including 1 group of commonly upregulated genes and 2 groups of commonly downregulated genes, were retrieved from the 3 planthoppers (Table S17). The protein lengths of these homologous genes ranged from 120 to 472 amino acids. We used these proteins as queries to search the NR database and found no homologous genes in other species with a cutoff of 1E^−7^, indicating that these genes are likely planthopper-specific genes.

Differences in immune response to virus infection in the 3 planthoppers were also observed. The RNAi pathway genes, RISC-loading complex TARBP2 and argonaute-3, were upregulated in *S. furcifera* and *N. lugens*, respectively, but genes in the RNAi pathway did not respond to virus infection in *L. striatellus*. The antimicrobial peptide defensin was upregulated in *L. striatellus* and *N. lugens* but was downregulated in *S. furcifera*. The expression of the Down syndrome cell adhesion molecule gene increased in *L. striatellus* [55] and decreased in *S. furcifera*, but did not show significant change in *N. lugens* in response to their respective plant viruses.

In summary, we reported a high-quality of genome of *L. striatellus*, a notorious rice pest insect. *L. striatellus* has the smallest genome and the lowest number of protein-coding genes compared with the other 2 rice planthoppers, *S. furcifera* and *N. lugens*. Comparative genomic analyses identified expansions and contractions in olfactory genes, detoxification genes, immune genes, and energy metabolism genes among the 3 rice planthoppers, which may have contributed to their differences in important traits such as host range, migratory habit, and plant virus transmission. Despite having the smallest genome, *L. striatellus* has the widest host plant range among the 3 planthoppers. This situation is different from that of the genome evolution in Aphididae, where the soybean aphid, *Aphis glycines*, which is an extreme specialist, has the smallest genome compared with another 3 aphid species with published genome sequences [56]. With the addition of the *L. striatellus* genome, the genome data of the 3 rice planthoppers will aid studies in various areas of planthoppers and promote control strategies in the future.

## Availability of supporting data

Genome sequencing and transcriptome data used for genome assembly and gene annotation are deposited in the SRA under bioproject number PRJNA393384. Further supporting data, including annotations, gene expression data, alignments, and BUSCO results, are available via the *GigaScience* repository, *Giga*DB (GigaDB, RRID:SCR_004002) [57].

## Abbreviations

BUSCO: benchmarking universal single-copy ortholog; CSP: chemosensory protein; DEG: differentially expressed gene; GO: gene ontology; GR: gustatory receptor; IR: ionotropic receptor; KEGG: Kyoto Encyclopedia of Genes and Genomes; OBP: odorant binding protein; OR: odorant receptor; RBSDV: rice black-streaked dwarf virus; RRSV: rice ragged stunt virus; RSV: rice stripe virus; SRBSDV: Southern rice black streak dwarf virus; TE: transposable element.

## Additional file

Table S1. Base composition of the *Laodelphax striatellus* genome assembly.

Table S2. Summary of reads mapping to the genome assembly of *Laodelphax striatellus*.

Table S3. Transcript-based evaluation of the genome assembly of *Laodelphax striatellus*.

Table S4. Statistics of 9 transcriptomic reads mapped to different genomic regions.

Table S5. Genome completeness assessment using benchmarking universal single copy orthologs in 5 insects.

Table S6. Repetitive elements predicted by different programs.

Table S7. Sources of genome data of 22 arthropod species.

Table S8. Gene models predicted by different methods.

Table S9. Statistical comparison of gene sets of *Laodelphax striatellus* and 9 other arthropod species.

Table S10. Expanded gene families in the 3 planthoppers.

Table S11. Chemoreception-related genes in the 3 planthoppers.

Table S12. Detoxification-related genes in the 3 planthoppers.

Table S13. Immune genes in the 3 planthoppers.

Table S14. Sources of gene annotation files for the 3 planthoppers.

Table S15. Shared gene ontology terms for differentially expressed genes in the 3 planthoppers responding to plant viruses.

Table S16. Commonly regulated genes with similar functions in the 3 planthoppers responding to plant viruses.

Table S17. Homologous genes in the 3 planthoppers responding to plant viruses.

Figure S1. *Laodelphax striatellus* genome size estimation by flow cytometry and k-mer analyses. (A), (B), and (C) Fluorescence peaks for *Drosophila melanogaster, Gallus gallus*, and *L. striatellus*, respectively. The genome sizes of *D. melanogaster* and *G. gallus* were 0.18 pg and 1.25 pg, respectively. The genome size of *L. striatellus* was calculated to be 0.60 pg. (D) The depth distribution of k-mers (k = 17).

Figure S2. *Laodelphax striatellus* chromosomes dyed with Hoechst 33 258. (A) Haploid chromosomes. (B) Diploid chromosomes.

Figure S3. Sequencing depth distribution. The x-axis shows sequencing depth, and the y-axis shows fraction of bases with certain sequencing depth.

Figure S4. Summary of gene structures of *Laodelphax striatellus* and 8 other species used for gene annotation.

Figure S5. BUSCO assessment of the *Laodelphax striatellus* gene set. The completeness of the gene set was assessed with 2 BUSCO v. 2 datasets (arthropoda and eukaryote). The recovered matches are classified as “complete” if their lengths are within the expectation of the BUSCO profile match lengths. If these are found only once, they are classified as “complete single,” and other “complete” matches are classified as “complete duplicated.” The matches that are only partially recovered are classified as “fragmented,” and BUSCO groups for which there are no matches that pass the tests of orthology are classified as “missing.” For each species, the right bar shows the arthropoda results and the left bar shows the eukaryote results. Aga: *Anopheles gambiae*; Agl: *Anoplophora glabripennis*; Ame: *Apis mellifera*; Api: *Acyrthosiphon pisum*; Bmo: *Bombyx mori*; Bta: *Bemisia tabaci*; Cle: *Cimex lectularius*; Dci: *Diaphorina citri*; Dme: *Drosophila melanogaster*; Dno: *Diuraphis noxia*; Dpl: *Danaus plexippus*; Dpu: *Daphnia pulex*; Lmi: *Locusta migratoria*; Lst: *Laodelphax striatellus*; Nlu: *Nilaparvata lugens*; Nvi: *Nasonia vitripennis*; Ofa: *Oncopeltus fasciatus*; Phu: *Pediculus humanus*; Rpr: *Rhodnius prolixus*; Sfu: *Sogatella furcifera*; Tca: *Tribolium castaneum*; Zne: *Zootermopsis nevadensis*.

Figure S6. Determination of genomic heterozygosity. (A) Density distribution of heterozygous rates. (B) Frequency distribution of read coverage of both high and low heterozygosity. All heterozygosity rates were ranked, and the top 20% were chosen as high heterozygosity (high_het in the legend) and the remainder as low heterozygosity (low_het in the legend).

Figure S7. Divergence times estimation of 22 arthropod species. The number on each node stands for the divergence time from the present (million years ago [Mya]), with 95% confidence interval values noted in brackets. Four calibration times were used in the estimation: *D. pulex–D. melanogaster* divergence (445∼530 Mya), *N. vitripennis*–*D. melanogaster* divergence (279∼306 Mya), *A. gambiae*–*D. melanogaster* divergence (235∼269 Mya), and *A. mellifera–N. vitripennis* divergence (175∼215 Mya). The location of *L. striatellus* was indicated by an arrow.

Figure S8. Gene family expansion and contraction in the 3 planthoppers. *R. prolixus* was used as an outgroup to construct the phylogenetic tree and infer expanded/contracted gene families by CAFÉ. A conditional *P*-value was calculated for each gene family, and families with *P*-values <0.05 were considered significantly expanded (green) or contracted (red).

## Competing interests

The authors declare that there are no financial and nonfinancial competing interests in this study.

## Funding

This work was supported by the Strategic Priority Research Program of the Chinese Academy of Sciences (No. XDB11040200), Major State Basic Research Development Program of China (973 Program; No. 2014CB13840402), and Natural Science Foundation of China (No. 31371934).

## Author contributions

J.Z. and F.J. collected the samples, prepared the DNA and RNA, analyzed the data, and drafted the paper. X.W. and P.Y. coordinated the project. Y.B. sequenced the transcriptomes. W.Z., W.W., H.L., Q.W., N.C., J.L., X.C., L.L., and J.Y. analyzed the data. L.K. and F.C. designed the research and wrote and revised the paper.

## Supplementary Material

GIGA-D-17-00204_Original-Submission.pdf

GIGA-D-17-00204_Revision-1.pdf

Response-to-Reviewer-Comments_Original-Submission.pdf

Reviewer-1-Report-(Original-Submission) -- Denis Tagu 23 Aug 2017 Reviewed

Reviewer-1-Report-(Revision-1) -- Denis Tagu 26 Sep 2017 Reviewed

Reviewer-2-Report-(Original-Submission) -- Spencer Johnston 19 Sep 2017 Reviewed

Additional Files
