# Supplementary material for: Genome sequence of the small brown planthopper, Laodelphax striatellus
Source: Gigascience. 2017 Nov 10;6(12):1–12. doi: 10.1093/gigascience/gix109 (PMC5740986; doi:10.1093/gigascience/gix109)
Supplement: GIGA-D-17-00204_Original-Submission.pdf [file gix109_giga-d-17-00204_original-submission.pdf]

GigaScience

Genome sequence of the small brown planthopper Laodelphax striatellus

--Manuscript Draft--

**Manuscript Number:**	GIGA-D-17-00204
**Full Title:**	Genome sequence of the small brown planthopper Laodelphax striatellus
**Article Type:**	Data Note
**Funding Information:**	Strategic Priority Research Program of the Chinese Academy of Sciences (XDB11040200) — Dr. Feng Cui<br>Major State Basic Research Development Program of China (973 Program) (2014CB13840402) — Dr. Feng Cui<br>National Natural Science Foundation of China (31371934) — Dr. Yanyuan Bao
**Abstract:**	Background: Laodelphax striatellus Fallén (Hemiptera: Delphacidae) is one of the most destructive rice pests. L. striatellus is different from another two genome-released rice planthoppers, Sogatella furcifera and Nilaparvata lugens, in many biological characteristics, such as host range, dispersal capacity, and vectoring plant viruses. Deciphering the genome of L. striatellus will help understand the genetic basis of the biological differences among the three rice planthoppers.<br>Findings: 193 Gb Illumina data and 32.4 Gb Pacbio data were generated and used to assemble a high quality L. striatellus genome sequence, which is 541 Mb in length and has a contig N50 of 118 Kb and a scaffold N50 of 1.08 Mb. Annotated repetitive elements account for 25.7% of the genome. 17736 protein-coding genes were annotated, capturing 97.6% and 98% of BUSCO eukaryote and arthropoda genes, respectively. Compared to N. lugens and S. furcifera, L. striatellus has the smallest genome and the least gene number. Gene family expansion and transcriptomic analyses provided hints to the genomic basis of the differences in important traits such as host range, migratory habit and plant virus transmission between L. striatellus and the other two planthoppers.<br>Conclusions: We reported a high quality genome assembly of L. striatellus, which is an important genomic resource not only for the study of the biology of L. striatellus and its interactions with plant hosts and plant viruses, but also for the comparisons to other planthoppers.
**Corresponding Author:**	Feng Cui<br>Institute of Zoology Chinese Academy of Sciences<br>Beijing, CHINA
**Corresponding Author Secondary Information:**	
**Corresponding Author's Institution:**	Institute of Zoology Chinese Academy of Sciences
**Corresponding Author's Secondary Institution:**	
**First Author:**	Junjie Zhu
**First Author Secondary Information:**	
**Order of Authors:**	Junjie Zhu
	Feng Jiang
	Xianhui Wang
	Pengcheng Yang
	Yanyuan Bao
	Wan Zhao
	Wei Wang

Powered by Editorial Manager® and ProduXion Manager® from Aries Systems Corporation

	Hong Lu
	Qianshuo Wang
	Na Cui
	Jing Li
	Xiaofang Chen
	Lan Luo
	Jinting Yu
	Le Kang
	Feng Cui
**Order of Authors Secondary Information:**	
**Opposed Reviewers:**	
**Additional Information:**	
**Question**	**Response**
Are you submitting this manuscript to a special series or article collection?	No
**Experimental design and statistics**<br><br>Full details of the experimental design and statistical methods used should be given in the Methods section, as detailed in our Minimum Standards Reporting Checklist. Information essential to interpreting the data presented should be made available in the figure legends.<br><br>Have you included all the information requested in your manuscript?	Yes
**Resources**<br><br>A description of all resources used, including antibodies, cell lines, animals and software tools, with enough information to allow them to be uniquely identified, should be included in the Methods section. Authors are strongly encouraged to cite Research Resource Identifiers (RRIDs) for antibodies, model organisms and tools, where possible.<br><br>Have you included the information requested as detailed in our Minimum Standards Reporting Checklist?	Yes
**Availability of data and materials**<br><br>All datasets and code on which the	Yes

conclusions of the paper rely must be either included in your submission or deposited in publicly available repositories (where available and ethically appropriate), referencing such data using a unique identifier in the references and in the "Availability of Data and Materials" section of your manuscript.<br><br>Have you have met the above requirement as detailed in our Minimum Standards Reporting Checklist?	

Powered by Editorial Manager® and ProduXion Manager® from Aries Systems Corporation

Genome sequence of the small brown planthopper *Laodelphax striatellus*

Junjie Zhu[1,4*], Feng Jiang[2*], Xianhui Wang[1], Pengcheng Yang[2], Yanyuan Bao[3], Wan Zhao[1], Wei Wang[1], Hong Lu[1], Qianshuo Wang[1], Na Cui[1], Jing Li[1], Xiaofang Chen[1], Lan Luo[1], Jinting Yu[1], Le Kang[1#], Feng Cui[1#]

[1]State Key Laboratory of Integrated Management of Pest Insects and Rodents, Institute of Zoology, Chinese Academy of Sciences, Beijing 100101, China

[2]Beijing Institutes of Life Science, Chinese Academy of Sciences, Beijing 100101, China

[3]State Key Laboratory of Rice Biology and Ministry of Agriculture Key Laboratory of Agricultural Entomology, Institute of Insect Sciences, Zhejiang University, Hangzhou 310058, China.

[4]University of Chinese Academy of Sciences, Beijing 100049, China

Junjie Zhu (zjj19910@163.com), Feng Jiang (jiangf@mail.biols.ac.cn), Xianhui Wang (wangxh@ioz.ac.cn), Pengcheng Yang (yangpc@mail.biols.ac.cn), Yanyuan Bao (yybao@zju.edu.cn), Wan Zhao (zhaow@ioz.ac.cn), Wei Wang (031150796@163.com), Hong Lu (creach2009@163.com), Qianshuo Wang (wqsh24@126.com), Na Cui (cuina@ioz.ac.cn), Jing Li (lj5213318@163.com), Xiaofang Chen (13163256118@163.com), Lan Luo (luolan@ioz.ac.cn), Jinting Yu

(13053277282@163.com), Le Kang (lkang@ioz.ac.cn), Feng Cui (cuif@ioz.ac.cn)

#Authors for correspondence:

Feng Cui

Tel: +86-10-64807218; Email: cuif@ioz.ac.cn.

Le Kang

Tel: +86-10-64807219; Email: lkang@ioz.ac.cn.

*These authors contributed equally to this work.

Abstract

Background: *Laodelphax striatellus* Fallén (Hemiptera: Delphacidae) is one of the most destructive rice pests. *L. striatellus* is different from another two genome-released rice planthoppers, *Sogatella furcifera* and *Nilaparvata lugens*, in many biological characteristics, such as host range, dispersal capacity, and vectoring plant viruses. Deciphering the genome of *L. striatellus* will help understand the genetic basis of the biological differences among the three rice planthoppers.

Findings: 193 Gb Illumina data and 32.4 Gb Pacbio data were generated and used to assemble a high quality *L. striatellus* genome sequence, which is 541 Mb in length and has a contig N50 of 118 Kb and a scaffold N50 of 1.08 Mb. Annotated repetitive elements account for 25.7% of the genome. 17736 protein-coding genes were annotated, capturing 97.6% and 98% of BUSCO eukaryote and arthropoda genes, respectively. Compared to *N. lugens* and *S. furcifera*, *L. striatellus* has the smallest genome and the least gene number. Gene family expansion and transcriptomic analyses provided hints to the genomic basis of the differences in important traits such as host range, migratory habit and plant virus transmission between *L. striatellus* and the other two planthoppers.

Conclusions: We reported a high quality genome assembly of *L. striatellus*, which is an important genomic resource not only for the study of the biology of *L. striatellus* and its interactions with plant hosts and plant viruses, but also for the comparisons to other planthoppers.

Keywords: *Laodelphax striatellus*; Planthopper; Genome assembly; Annotation; Virus transmission; *Sogatella furcifera*; *Nilaparvata lugens*

Data description

The small brown planthopper, *Laodelphax striatellus* (Delphacidae, Hemiptera), is one of the most destructive pests in a variety of crops (Figure 1). It is widespread in Palearctic region, including countries such as China, Japan, Germany, Italy, Russia, Kazakhstan, Turkey, and United Kingdom [1]. *L. striatellus* is polyphagous and its hosts include rice, maize, oats, tall oatgrass, wheat, and barley. It injures plants by sap-sucking behavior using its piercing-sucking mouthpart, after which symptoms like stunting, chlorosis and hopper burn may further develop in plants. Apart from feeding damage, *L. striatellus* transmits various plant viruses, such as *Rice stripe virus* (RSV), *Rice black-streaked dwarf virus* (RBSDV), *Barley yellow striate mosaic virus*, *Maize rough dwarf virus*, *Wheat rosette stunt virus*, and *Wheat chlorotic streak virus* [2]. Some of these viruses may cause serious damage to agricultural production, such as RSV and RBSDV. For example, rice stripe disease caused by RSV has outbroken over the past several decades in many East Asian countries, including China, where rice field production was reduced by 30%–50% and total loss of harvest was observed in some areas [3].

L. striatellus is distinct from another two rice planthoppers, white-backed planthopper (*Sogatella furcifera*) and brown planthopper (*Nilaparvata lugens*), in several important traits such as host range, dispersal capacity, and plant viruses that they vector. *N. lugens* mostly feeds on rice plants, *S. furcifera* feeds on rice, wheat and maize, and *L. striatellus* has an even broader host range. Both *N. lugens* and *S. furcifera* are known for migratory habits [4]. Whereas, *S. furcifera* is the vector of *Southern rice*

black streak dwarf virus (SRBSDV) [5], and *N. lugens* is the vector of *Rice ragged stunt virus* (RRSV) and *Rice grassy stunt virus* [6, 7], *L. striatellus* is the carrier of RSV, RBSDV and several other viruses. Although the genome sequences of *S. furcifera* and *N. lugens* have been released recently [8, 9], no comparative genomic analyses were reported for the two planthoppers. Deciphering the genome of *L. striatellus* can help understand the genetic basis underlying the differences in important traits between *L. striatellus* and the other two rice planthoppers.

Sample and sequencing

The inbreeding line used for genome sequencing is an inbred laboratory strain that was derived from a field population collected in Hai'an, Jiangsu province, China. A single gravid female was selected and her progenies were sib-mated for 22 generations to obtain the inbreeding line. Planthoppers were reared on 2-3 cm rice seedlings at 25 °C and a photoperiod of 16:8 h light/dark. DNA was extracted by using Puregene Core Kit A (Qiagen) from the F22 specimens following the manufacturer's instruction. We built 5 libraries with insert size between 180 bp and 800 bp for paired-end sequencing and 9 libraries with insert size between 1.4 Kb to 24 Kb for mate-pair sequencing according to standard protocols of Illumina HiSeq 2500 sequencer (Table 1). We also constructed 33 Pacbio RSII libraries according to the standard Pacbio protocols (Table 1). Totally We generated 190 Gb Illumina data (126 Gb paired-end reads and 64 Gb mated-pair reads) and 32.4 Gb Pacbio data, representing 316X and 54X coverage of the genome, respectively.

For transcriptome sequencing, total RNAs were isolated from four tissues (antenna, brain, fatty body and gonad) and whole bodies of three developmental stages (egg, nymph, adult) of *L. striatellus* using TRIzol reagent (Invitrogen) according to the manufacturer's protocol. Nanodrop (Thermo Scientific) was used to determine RNA quantity and gel electrophoresis was used to examine RNA quality. cDNA libraries were constructed according to the manufacturer's instructions and sequenced on an Illumina HiSeq 2500 sequencer.

Estimation of genome size and determination of chromosome number

We estimated the genome size of *L. striatellus* using two independent approaches: flow cytometry [8] and *k*-mer analyses [10]. The flow cytometry analysis was carried out according to a published procedure [8]. Briefly, a female adult was ground in the PBS-T buffer. The mixture was filtered by a 40 μm cell filter, incubated with 2 μg/ml RNase A at 37 °C for 15 minutes and then stained with 5 μg/ml propidium iodide at 25 °C for 30 minutes. The fluorescence signal was detected by a FACSCallbur Analyzer (Becton, Dickinson and Company). Heads of *Drosophila melanogaster* and cytoblasts of *Gallus gallus* were treated with the same procedure as genome size references. The genome sizes of *D. melanogaster* and *G. gallus* are known to be 0.18 pg and 1.25 pg, respectively [11]. As shown in Figure S1, the genome size of *L. striatellus* was estimated to be 0.60 pg (587 Mb) by the flow cytometry method. In *k*-mer analysis, 31.94 Gb clean reads were utilized to generate a *k*-mer (k = 17) depth distribution curve (Figure S1D), based on which the genome size was estimated to be 550 Mb.

Accordingly, the haploid genome size of *L. striatellus* was estimated to be 550-587 Mb.

The chromosome number was determined by cytological analysis of testes cells. The testes of newly emerged males were dissected in insect Ringer solution. After fixed in Carnoy's fixative for 15 minutes, the testes were washed with 0.01 mol/L PBS solution, stained at 0.5 μg/ml Hoechst 33258, and sealed with Antifade Mounting Medium (Beyotime). Cells in meiosis phase were selected for chromosome counting under a confocal microscope Zeiss LSM710 (Zeiss). In most cases, 15 haploid chromosomes were observed (30 for diploid chromosomes, Figure S2), although sometimes only 14 were visible. Thus the number of chromosomes in *L. striatellus* was determined to be 2n = 30.

Genome assembly and assessment

We assembled the genome with both Illumina sequencing and Pacbio sequencing data. Illumina data were used to build contigs and scaffolds as follows. First, all reads with >= 10% unidentified nucleotides, or with > 10 nt aligned to the adapter sequences, or being putative PCR duplicates were removed to obtain clean reads. Mate-pair reads from libraries with insert sizes >2 kb were classified as paired-end, unpaired, negative, and mate-pair reads and only the negative and mate-pair reads were retained for the assembly. Second, we employed SOAPdenovo (Ver. 3.0) [12, 13] with the parameters "pregraph -K 33 -p 30 -d 30; contig –k 33 –M 3" to build de Bruijn graph and assemble sequencing reads into contigs. Third, all mate-pair reads were mapped to the contigs and mate-pair information was added in a stepwise manner to connect contigs into

scaffolds. GapCloser (Ver. 1.12) [12] was used to fill the gaps between scaffolds with a local assembly strategy. Afterwards, PBJelly (Ver. 15.8.24) [14] was used to fill gaps between scaffolds using the 32.4 Gb (~ 54X) Pacbio data. Briefly, all the gaps (length >25 bp) on the assembly were identified first and the Pacbio reads were mapped to the assembly using PBJelly. The BLASR alignments were parsed to identify gap-supporting reads by comparing aligned and un-aligned base positions within each read [15]. Overlap-layout-consensus engine ALLORA within PBSuite (Ver. 15.8.24, Pacific Biosciences Menlo Park) [16] was used to assemble the reads for each gap to generate consensus gap-filling sequences. As the final step, the consensus gap filling sequences were spliced into the corresponding gap position in the draft assembly, replacing all N's if the gap was closed and leaving appropriate number of N's if the gap was only reduced.

With the above assembly procedure, we obtained a final assembly of 541 Mb, having 38,193 scaffolds with a contig N50 length of 118 Kb and a scaffold N50 length of 1.1 Mb. The length of the assembly accounts for 91.7% and 98.4% of the estimated genome size by flow cytometry and *k*-mer analysis, respectively. The longest contig and scaffold were 2.0 Mb and 10.4 Mb, respectively (Table 2). The Pacbio sequencing data greatly improved the length of contigs compared to the published genomes of *N. lugens* (contig N50, 24.2 Kb [8]) and *S. furcifera* (contig N50, 70.7 Kb [9]), which were assembled with Illumina data only (Table 2). We aligned clean reads onto the genome assembly using BWA [17] and calculated the fraction of bases at given sequencing depth. The results showed a very small fraction of low coverage bases, suggesting high coverage and accuracy of the genome assembly (Figure S3).

The completeness and accuracy of the genome assembly were assessed by four independent approaches. First, the base compositions of each bin were calculated. As shown in Table S1, the assembled genome had a proper base composition and low percentage (1.99%) of Ns. The GC content of *L. striatellus* was 34.54%, similar to that of *N. lugens* [8] and slightly higher than that of *S. furcifera* [9]. Second, we remapped Illumina paired-end reads to the assembly using BWA [17] and we found that 93.2% reads could be mapped back, covering 96.83% of the assembled genome, including 95.08% of the genome with ≥ 20X coverage (Table S2). Third, we performed *de novo* transcriptome assembly using Trinity (Ver. 2.0.2) for RNA-seq data from multiple developmental stages and tissues (Table 1). We also included two published RNA sequencing datasets from salivary glands and alimentary canal [18] in the transcriptome assembly. We mapped the assembled transcripts to the genome assembly using Tophat with default parameters and found that 90.31% of the transcripts with > 90% transcript coverage were aligned to one scaffold (Table S3), indicating that most expressed genes were correctly assembled in the genome. When the RNA reads from the nine transcriptome datasets were directly mapped to the genome, 78% to 94% were could be correctly mapped to the genome with appropriate splicing, indicating that the genome assembly had a good representative of gene regions (Table S4). Finally, the benchmarking universal single-copy orthologs (BUSCO, Ver. 1) dataset representing 2675 genes for arthropoda was used for genome assessment [19]. Our assembled genome captured 92% (2470/2675) of the BUSCO genes, suggesting that a gene repertoire was nearly complete (Table S5). Taken together, these results suggest that

our assembled genome was highly accurate and nearly covered the whole genome.

Annotation of repetitive elements

Two independent methods, namely homology-based and *de novo* prediction, were applied for repetitive element annotation. For the homology-based method, the assembled genome was compared to Repbase issued on January 13, 2014 [20] using RepeatMasker (Ver. 4.0.5) and RepeatProteinMasker (Ver. 1.36) with default settings [21]. For the *de novo* prediction, we built a *de novo* repeat library with LTR_FINDER (Ver. 1.0.5) [22], Piler (Ver. 1.06) [23], RepeatScout (Ver. 1.0.5) [24] and RepeatModeler (Ver. 1.0.8). Tandem Repeat Finder (Ver. 4.07b) [25] was used to search tandem repeats. Furthermore, RepeatProreinMask [21] was used to identify putative transposable element (TE) related proteins. After merging all the repetitive elements identified by above-mentioned tools, we identified a total of 139.1 Mb repetitive sequences, accounting for 25.7% of the genome (Table S6). The percentage of repetitive elements in the *L. striatellus* genome was much lower than those of *N. lugens* (48.6% [8]) and *S. furcifera* (44.3% [9]). Of all the repetitive sequences, 10.59% were the class I transposable elements (retrotransposon), including 5.01% long interspersed nuclear elements, 1.32% long terminal repeats, and 4.26% short interspersed nuclear elements. Class II elements (DNA transposons) represented only 4.92% of the genome (Table 3). *L. striatellus* had the lowest TE fraction and the smallest genome size compared to *N. lugens* and *S. furcifera* (Table 3).

Annotation of protein-coding genes

The protein-coding genes were annotated with evidences from the homology-base method, *ab initio* prediction, and RNA-seq data. For the homology-based method, the annotated gene sets from eight species, *N. lugens*, *Acyrthosiphon pisum*, *Pediculus humanus*, *Nasonia vitripennis*, *D. melanogaster*, *Bombyx mori*, *Rhodnius prolixus* and *Daphnia pulex* (Table S7) were aligned to the *L. striatellus* genome using TBLASTN [26] with an E-value cutoff of $1E^{-5}$. Genewise (Ver. 2.2.0) [27] was used to define gene models. For *ab initio* prediction, we utilized Augustus (Ver. 3.1) [28], GlimmerHMM (Ver. 3.0.4) [29], SNAP (Ver. 2013-11-29) [30], GeneID (Ver. 1.4) [31, 32] and Genescan (Ver. 1.0) [33] to predict potential protein-coding genes from the repeat-masked genome. Furthermore, we identified gene structures with the assistance of nine transcriptomes assembled by Tophat-Cufflinks (Ver. 2.2.1) [34] and Trinity-PASA (Ver. 2.0.2) [35], respectively. Then we integrated all predicted gene structures above with EvidenceModeler (Ver. 1.1.1) [36] was used to integrate all predicted gene structures from above-mentioned procedures to obtain a non-redundant set of 17736 protein-coding genes with an average gene length of around 16.17 Kb (Table S8-S9, Figure S4). We constructed the orthologous gene families using annotated genes from 22 closely-related species (Table S7) and found that *L. striatellus* had 4210 species-specific genes, much fewer than those of *N. lugens* (10163) and *S. furcifera* (7743) (Figure 2). This may be attributed to the smaller genome size and lower gene number in *L. striatellus*.

We used three methods to evaluate the gene models that we obtained. First, we examined the 2 Kb upstream and downstream regions of annotated genes and found

that the majority (16525, 93.17%) of genes did not contain any ambiguous bases (Ns) in the 2 Kb up- and down-stream regions, indicating that these gene models are not located near an assembly gap and thus the gene models are unlikely to be a fragment. Second, we compared our annotated genes with the corresponding orthlogous genes in *D. melanogaster*. We performed BLASTX [26] searches against the *D. melanogaster* gene set using the *de novo* assembled transcripts in *L. striatellus*. A total of 8484 assembled transcripts that had identity > 60% with a *D. melanogaster* gene and covered > 90% of the coding region were regarded as full-length transcripts. Among them, 3728 transcripts (excluding redundant protein isoforms) containing a complete ORF were searched against the annotated genes and 3093 (82.97%) of them had near perfect match to an annotated gene, indicating that most annotated genes were complete. Third, we compared our annotated genes to the two sets of BUSCO (Ver. 2) genes (1066 arthropoda genes and 303 eukaryote genes) [19] and found that our predicted genes were considered as complete BUSCO genes in 97.6% and 98.0% of the eukaryote genes and arthropoda genes, respectively (Figure S5), suggesting that a nearly complete repertoire of protein-coding gene set was determined.

To estimate the level of heterozygosity in the gene model, we aligned 23X reads to the genome assembly with BWA [17]. After removing duplicates, heterozygous SNPs were identified using BCFtools [37]. The heterozygous SNPs in the coding regions of each gene were used to compute read coverage and heterozygosity. Only a single heterozygosity peak of around 0.3 was detected (Figure S6A). We ranked the heterozygosity rate of all the gene set and took the top 20% as high heterozygosity (the

left as low heterozygosity). Coverage histograms of high and low heterozygosity showed similar range of coverage distribution (Figure S6B). Therefore, the heterozygosity did not influence the gene annotation.

In order to obtain putative functional assignments to the annotated genes, we compared the annotated protein sequences of *L. striatellus* to proteins in KEGG [38], NR [39] and Swiss-Prot [40] databases using BLASTP [26] with an E-value cutoff of $1E^{-5}$. Domains and motifs were scanned in Interpro [41] database by InterProScan [42]. There were 78.7%, 66.3%, 63.6%, and 69.5% of annotated proteins showing significant sequence similarity with the proteins in NR, Swiss-prot, KEGG, and InterPro, respectively. Among the 12322 genes with an InterPro hit, 11159 (90.6%) had Pfam annotations and 8935 (72.5%) had Gene Ontology (GO) associations. After removing redundancy, 14182 of 17736 genes (80.0%) were assigned to known databases (Figure 3). Among the 3554 unannotated genes, 1391 (7.8%) were *L. striatellus*-specific genes.

Gene orthology prediction

Twenty one sequenced insects (*Zootermopsis nevadensis*, *Tribolium castaneum*, *Anoplophora glabripennis*, *Anopheles gambiae*, *D. melanogaster*, *A. pisum*, *Diuraphis noxia*, *Cimex lectularius*, *L. striatellus*, *R. prolixus*, *N. lugens*, *S. furcifera*, *Diaphorina citri*, *Oncopeltus fasciatus*, *Apis mellifera*, *N. vitripennis*, *B. mori*, *B. tabaci*, *Danaus plexippus*, *Locusta migratoria*, and *P. humanus*) and one non-insect arthropoda sequenced species (*D. pulex*) were used to infer gene orthology and reconstruct the phylogenetic tree. The annotated coding sequences were downloaded from the websites

listed in Table S7. The homologous gene families were identified using TreeFam [43, 44] and ascribed in different categories (Figure 2). The gene families were identified following these steps: i) BLASTP [26] was used to compare all protein sequences for the 22 species with an E-value cutoff of $1E^{-7}$; ii) the blast alignments were concatenated by Solar (Ver. 0.9.6) [44], followed by homology identification among protein sequences; and iii) gene families were identified using hcluster_sg (Ver. 0.5.0) [44]. RAxML (Ver. 8.0.19) [45] was used to reconstruct the phylogenetic tree based on the concatenated single-copy protein sequences under PROTGAMMAAUTO model with 100 bootstrap replicates. R8s (Ver. 1.7.1) [46] and MCMCtree (PAML package, Ver. 4.7) [47] were used to estimate the divergence times among species. The parameters used in MCMCtree were "--rootage 510 -clock 3 -alpha 0.977999 -model 7". To examine gene family expansion and contraction in the three planthoppers, we chose one additional hemipteran species *R. prolixus* as outgroup to infer expanded/contracted gene families using CAFE (Ver. 3.1) [48]. A conditional *P*-value was calculated for each gene family and the gene families with *P*-value < 0.05 were considered as significantly expanded or contracted. The phylogenetic analysis revealed that *L. striatellus* clustered together with the other two planthoppers and had a closer relationship to *S. furcifera* than *N. lugens* (Figure 4). The divergence times of non-planthopper insect species were generally consistent with those estimated in the previous study [8]. The result of molecular dating analysis indicated that the ancestor of *L. striatellus* and *S. furcifera* split with *N. lugens* about 87.5 million years ago and *L. striatellus* diverged from *S. furcifera* approximately 31 million years ago (Figure S7).

Compared with *N. lugens* and *S. furcifera*, *L. striatellus* had fewer expanded gene families and more contracted gene families (Figure S8). This might partially explain why *L. striatellus* has the fewest gene number among the three planthopper species. Since the divergence of *L. striatellus* and *S. furcifera*, *L. striatellus* and *S. furcifera* had 95 and 547 expanded gene families, respectively (Figure S8). The significantly expanded gene families in *L. striatellus* included some specific members of multi-gene families, such as odorant receptor, cytochrome P450, and serine protease (especially trypsin, Table S10). The specific members of chemosensory protein, odorant binding protein, carboxylesterase, and ATP-binding cassette transporter families were also increased in *L. striatellus* although their *P*-values were higher than 0.05 (Table S10). Expansion of these gene families may have contributed to the widest host plant range of *L. striatellus* among the three planthoppers. The specific members of gene families associated with energy metabolism were significantly expanded in *S. furcifera*, such as acyl-CoA synthetase, fatty acyl-CoA reductase, acyl-CoA-binding protein, and acyl-coenzyme A thioesterase. The specific members of glyceraldehyde-3-phosphate dehydrogenase, D-beta-hydroxybutyrate dehydrogenase, ADP/ATP translocase, acyl-CoA transporter, and ATP synthase families also increased, even though with *P*-values higher than 0.05 (Table S10). *N. lugens* had 433 expanded gene families (Figure S8). A bunch of specific members from energy metabolism related gene families, including Delta(3,5)-Delta(2,4)-dienoyl-CoA isomerase, ATP-citrate synthase, malonyl-CoA decarboxylase, NADH dehydrogenase (ubiquinone) 1α subcomplex subunit 7 and subunit 8, acyl-CoA synthetase, ATP synthase, and enoyl-CoA delta isomerase

increased in *N. lugens* although their *P*-values were higher than 0.05 (Table S10). Expansion in the energy metabolism related gene families is in accordance with the migratory habit of *S. furcifera* and *N. lugens*.

Olfaction and detoxification system

It is essential for herbivorous insects to recognize and locate their host plants utilizing their sense of gustation and olfaction. Chemicals from the environment are received and recognized by chemoreceptor genes, including odorant receptors (ORs), gustatory receptors (GRs), and ionotropic receptors (IRs) in gustatory and olfactory organs. Detoxification gene families also play an essential role in defense against natural xenobiotics from host plants or synthetic xenobiotics including insecticides. To identify chemoreception and detoxification related genes in *L. striatellus*, we retrieved corresponding gene sequences of other insect species from previous studies and used as queries. These genes were searched against the *L. striatellus* gene set using BALSTP [26] with an E-value cutoff of $1E^{-5}$. In addition, we scanned the gene sets of three planthoppers for domain information using InterProScan and extracted genes with domains corresponding to each family. Finally we integrated results from both BLASTP and InterProScan to obtain the final set of protein families.

There were 106 ORs, 38 IRs, and 12 GRs identified in *L. striatellus* (Table S11). The numbers of OR and GR in *L. striatellus* were over twice as many as those in *N. lugens* and *S. furcifera*, representing a significant expansion in these two families. This is consistent with the fact that *L. striatellus* is the most polyphagous among the three

planthoppers because polyphagous insects tend to have more OR genes than monophagous [8]. Moreover, we identified two proteins families important for odor recognition and pheromone perception, namely odorant binding proteins (OBPs) and chemosensory proteins (CSPs). There were 16 OBPs and 31 CSPs in *L. striatellus*, the most among the three planthoppers (Table S11). The relatively higher number of odor related genes in *L. striatellus* might be closely related to its polyphagous habit.

We manually annotated families of detoxification related genes, including 26 UDP-glycosyltransferases, 29 glutathione-S-transferases, 54 carboxyl/cholinesterase, 73 ATP-binding cassette transporters, and 76 cytochrome P450s in *L. striatellus* (Table S12). The total number of detoxification related genes in *L. striatellus* was smaller than that in *N. lugens*, but larger than that in *S. furcifera*.

Immune-related genes

We identified immune gene repertoires of the three planthoppers based on a homology-based method. Immune genes from *D. melanogaster*, *A. gambiae*, *Aedes aegypti*, *Culex quinquefasciatus* were downloaded from ImmunoDB [49]. Gene sets from the three planthoppers were used as queries and searched against the immune genes of the four insects, respectively, using BLASTX with an E-value cutoff of $1E^{-5}$. The best hits were selected for further domain architecture analysis using InterProScan and then were confirmed manually. The number of immune-related genes in *L. striatellus* was 330, which was more than that in *N. lugens* (289) and *S. furcifera* (280) (Table S13). The redundant copies of immune genes in *L. striatellus* mainly included

autophagy genes, 1,3-beta-D glucan binding protein genes, clip-domain serine protease genes, and genes of small RNA regulatory pathway members. However, the numbers of C-type lectin genes and Toll-like receptor genes were fewer in *L. striatellus* compared to the other two planthoppers.

Transcriptomic responses of three planthoppers to their borne plant viruses

L. striatellus, *S. furcifera*, and *N. lugens* transmit different rice viruses. To explore the molecular response to respective plant viruses, we analyzed and compared the transcriptomic responses of *L. striatellus* to RSV, *S. furcifera* to SRBSDV, and *N. lugens* to RRSV. For *L. striatellus,* RSV was incubated in the 4th-instar nymphs for 5 d as described previously [50]. Three replicates of infected or non-infected insects were used to construct paired-end RNA-seq libraries for sequencing on an Illumina Hiseq 2500 sequencer. The transcriptomic data of *S. furcifera* infected with SRBSDV were retrieved from a previous study [51]. The 3rd-instar nymphs of *N. lugens* were infected by RRSV for 7 d before collected for RNA extraction using the SV Total RNA Isolation System (Promega). The gene expression libraries for RRSV-infected and non-infected samples were constructed and sequenced on an Illumina HiSeq 2000 sequencer. RNA-seq reads were mapped to the corresponding genome using TopHat2 (Ver. 2.1.1) [52]. For *L. striatellus* and *S. furcifera*, HTSeq [53] was used to count the number of reads mapped to each gene model, and the edgeR package was used to identify differentially expressed genes (DEGs) with a fold change cutoff of 2 and FDR cutoff of 0.01. For *N. lugens,* GFOLD (generalized fold change for ranking differentially expressed genes

from RNA-seq data) was used to detect DEGs without biological replicates. The gene annotation files were downloaded from the corresponding websites (Table S14). We referred genes with higher expressions in the viruliferous group as up-regulated genes and lower as down-regulated. The results showed that 460 (185 up and 275 down), 162 (48 up and 114 down), 1070 (515 up and 555 down) genes were differentially expressed in *L. striatellus*, *N. lugens* and *S. furcifera*, respectively, when bearing respective plant virus.

The DEGs in the three planthoppers were compared in GO terms and the common GO terms were retrieved (Table S15). The up-regulated genes in the three planthoppers were involved in the biological processes of regulation of transcription (GO:0006355) and protein phosphorylation (GO:0006468). The down-regulated genes in the three planthoppers took part in the biological processes of carbohydrate metabolic process (GO:0005975), chitin catabolic process (GO:0006032), and proteolysis (GO:0006508).

Two zinc finger proteins of *L. striatellus*, one zinc finger protein of *N. lugens*, and six zinc finger proteins of *S. furcifera* were commonly up-regulated while genes of chitinases, cytochrome P450 CYP4s, and trypsins were commonly down-regulated in the three planthoppers (Table S16) in response to respective plant virus. We also identified homologous genes that were commonly regulated in the three planthoppers by aligning *N. lugens* DEGs with those of *L. striatellus* and *S. furcifera* using BLASTP with a cutoff of $1E^{-3}$, a sequence identity higher than 60% and a coverage higher than 50%. Three groups of homologous genes, including one group of commonly up-regulated genes and two groups of commonly down-regulated genes, were retrieved

from the three planthoppers (Table S17). The protein lengths of these homologous genes ranged from 120 to 472 amino acids. We used these proteins as queries to search the NR database and found no homologous genes in other species with a cutoff of $1E^{-7}$, indicating that these genes are likely planthopper-specific genes.

Differences in immune response to virus infection in the three planthoppers were also observed. The RNAi pathway genes, RISC-loading complex TARBP2 and argonaute-3, were up-regulated in *S. furcifera* and *N. lugens*, respectively, but genes in the RNAi pathway did not respond to virus infection in *L. striatellus*. The antimicrobial peptide defensin was up-regulated in *L. striatellus* and *N. lugens* but was down-regulated in *S. furcifera*. The expression of Down syndrome cell adhesion molecule gene increased in *L. striatellus* [54], decreased in *S. furcifera*, but did not show significant change in *N. lugens* in response to respective plant viruses.

In summary, we reported a high quality of genome of *L. striatellus*, a notorious rice pest insect. *L. striatellus* has the smallest genome and the least number of protein-coding genes compared to the other two rice planthoppers, *S. furcifera* and *N. lugens*. Comparative genomic analyses identified expansions and contractions in olfactory genes, detoxification genes, immune genes, and energy metabolism genes among the three rice planthoppers, which may have contributed to their differences in important traits such as host range, migratory habit, and plant virus transmission. With the addition of the *L. striatellus* genome, the genome data of the three rice planthoppers will boost the studies in various areas of planthoppers and promote the control strategies in future.

Availability of supporting data

Genome sequencing and transcriptome data used for genome assembly and gene annotation are deposited in the SRA under bioproject number PRJNA393384. Further supporting data are available in GigaScience database.

List of abbreviations

BUSCO: benchmarking universal single-copy ortholog; CSP: chemosensory protein; DEG: differentially expressed gene; GO: Gene Ontology; GR: gustatory receptor; IR: ionotropic receptor; OBP: odorant binding protein; OR: odorant receptor; RBSDV: Rice black-streaked dwarf virus; RRSV: Rice ragged stunt virus; RSV: Rice stripe virus; SRBSDV: Southern rice black streak dwarf virus; TE: transposable element;

Competing interests

The authors declare that there are no financial and non-financial competing interests in this study.

Authors' contributions

JZ and FJ collected the samples, prepared the DNA and RNA, analyzed the data, and drafted the paper. XW and PY coordinated the project. YB sequenced the transcriptomes. WZ, WW, HL, QW, NC, JL, XC, LL and JY analyzed the data. LK and FC designed the research, wrote and revised the paper.

Acknowledgements

We thank Prof. Thomas Sicheritz-Pontén from Technical University of Denmark and Prof. Renyi Liu from Shanghai Center for Plant Stress Biology and Center of Excellence for Molecular Plant Sciences, Chinese Academy of Sciences for comments and language suggestions. This work was supported by the Strategic Priority Research Program of the Chinese Academy of Sciences (No. XDB11040200), Major State Basic Research Development Program of China (973 Program) (No. 2014CB13840402), and Natural Science Foundation of China (No. 31371934).

References

1. 3I Interactive Keys and Taxonomic Databases. Dmitriev DA. 2003. http://dmitriev.speciesfile.org/index.asp. Accessed 1 May 2017.
2. GENUS LAODELPHAX FENNAH, 1963. College of Agriculture & Natural Resources, University of Delaware. http://ag.udel.edu/research/delphacid/species/Laodelphax.htm. Accessed 1 May 2017.
3. Sun DZ and Jiang L. Research on the Inheritance and Breeding of Rice Stripe Resistance. Chinese Agricultural Science Bulletin. 2006; 12: 073.
4. Huang HJ, Xue J, Zhuo JC, Cheng RL, Xu HJ, and Zhang CX. Comparative analysis of the transcriptional responses to low and high temperatures in three rice planthopper species. Molecular ecology. 2017; 26(10): 2726-37.

5. Zhou GH, Wen JJ, Cai DJ, Li P, Xu DL, and Zhang SG. Southern rice black-streaked dwarf virus: a new proposed Fijivirus species in the family Reoviridae. Chinese Science Bulletin. 2008; 53(23): 3677-85.

6. Jia DS, Guo NM, Chen HY, Akita F, Xie LH, Omura T, et al. Assembly of the viroplasm by viral non-structural protein Pns10 is essential for persistent infection of rice ragged stunt virus in its insect vector. Journal of General Virology. 2012; 93(10): 2299-309.

7. Zheng LM, Mao QZ, Xie LH, and Wei TY. Infection route of rice grassy stunt virus, a tenuivirus, in the body of its brown planthopper vector, Nilaparvata lugens (Hemiptera: Delphacidae) after ingestion of virus. Virus Research. 2014; 188: 170-73.

8. Xue J, Zhou X, Zhang CX, Yu L-L, Fan HW, Wang Z, et al. Genomes of the rice pest brown planthopper and its endosymbionts reveal complex complementary contributions for host adaptation. Genome Biology. 2014; 15(12): 521.

9. Wang L, Tang N, Gao XL, Chang ZX, Zhang LQ, Zhou GH, et al. Genome sequence of a rice pest, the white-backed planthopper (Sogatella furcifera). GigaScience. 2017; 6(1): 1.

10. Li RQ, Fan W, Tian G, Zhu HM, He L, Cai J, et al. The sequence and de novo assembly of the giant panda genome. Nature. 2010; 463(7279): 311.

11. Bennett MD, Leitch IJ, Price HJ, and Johnston JS. Comparisons with Caenorhabditis (~ 100 Mb) and Drosophila (~ 175 Mb) using flow cytometry

show genome size in Arabidopsis to be~ 157 Mb and thus~ 25% larger than the Arabidopsis genome initiative estimate of~ 125 Mb. Annals of Botany. 2003; 91(5): 547-57.

12. Huang J, Zhang CM, Zhao X, Fei ZJ, Wan KK, Zhang Z, et al. The Jujube genome provides insights into genome evolution and the domestication of sweetness/acidity taste in fruit trees. PLOS Genetics. 2016; 12(12): e1006433.

13. Wang S, Zhang JB, Jiao WQ, Li J, Xun XG, Sun Y, et al. Scallop genome provides insights into evolution of bilaterian karyotype and development. Nature Ecology & Evolution. 2017; 1: 0120.

14. English AC, Richards S, Han Y, Wang M, Vee V, Qu JX, et al. Mind the gap: upgrading genomes with Pacific Biosciences RS long-read sequencing technology. PloS one. 2012; 7(11): e47768.

15. Chaisson MJ and Tesler G. Mapping single molecule sequencing reads using basic local alignment with successive refinement (BLASR): application and theory. BMC bioinformatics. 2012; 13(1): 238.

16. Kelley RK, Wang G, and Venook AP. Biomarker use in colorectal cancer therapy. Journal of the National Comprehensive Cancer Network. 2011; 9(11): 1293-302.

17. Li H and Durbin R. Fast and accurate short read alignment with Burrows–Wheeler transform. Bioinformatics. 2009; 25(14): 1754-60.

18. Zhao W, Lu LX, Yang PC, Cui N, Kang L, and Cui F. Organ-specific transcriptome response of the small brown planthopper toward rice stripe virus.

Insect Biochemistry and Molecular Biology. 2016; 70: 60-72.

19. Simão FA, Waterhouse RM, Ioannidis P, Kriventseva EV, and Zdobnov EM. BUSCO: assessing genome assembly and annotation completeness with single-copy orthologs. Bioinformatics. 2015; 31(19): 3210-12.

20. Bao WD, Kojima KK, and Kohany O. Repbase Update, a database of repetitive elements in eukaryotic genomes. Mobile DNA. 2015; 6(1): 11.

21. Tarailo- Graovac M and Chen NS. Using RepeatMasker to identify repetitive elements in genomic sequences. Current Protocols in Bioinformatics. 2009: 4.10. 1-4.10. 14.

22. Xu Z and Wang H. LTR_FINDER: an efficient tool for the prediction of full-length LTR retrotransposons. Nucleic Acids Research. 2007; 35(suppl_2): W265-W68.

23. Edgar RC and Myers EW. PILER: identification and classification of genomic repeats. Bioinformatics. 2005; 21(suppl_1): i152-i58.

24. Price AL, Jones NC, and Pevzner PA. De novo identification of repeat families in large genomes. Bioinformatics. 2005; 21(suppl_1): i351-i58.

25. Benson G. Tandem repeats finder: a program to analyze DNA sequences. Nucleic Acids Research. 1999; 27(2): 573.

26. Altschul SF, Gish W, Miller W, Myers EW, and Lipman DJ. Basic local alignment search tool. Journal of Molecular Biology. 1990; 215(3): 403-10.

27. Birney E, Clamp M, and Durbin R. GeneWise and genomewise. Genome Research. 2004; 14(5): 988-95.

28. Keller O, Kollmar M, Stanke M, and Waack S. A novel hybrid gene prediction method employing protein multiple sequence alignments. Bioinformatics. 2011; 27(6): 757-63.

29. Majoros WH, Pertea M, and Salzberg SL. TigrScan and GlimmerHMM: two open source ab initio eukaryotic gene-finders. Bioinformatics. 2004; 20(16): 2878-79.

30. Bedell JA, Korf I, and Gish W. MaskerAid: a performance enhancement to RepeatMasker. Bioinformatics. 2000; 16(11): 1040-41.

31. Blanco E and Abril JF. Computational gene annotation in new genome assemblies using GeneID. Bioinformatics for DNA Sequence Analysis. 2009: 243-61.

32. Blanco E, Parra G, and Guigó R. Using geneid to identify genes. Current Protocols in Bioinformatics. 2007: 4.3. 1-4.3. 28.

33. Burge C and Karlin S. Prediction of complete gene structures in human genomic DNA. Journal of Molecular Biology. 1997; 268(1): 78-94.

34. Trapnell C, Roberts A, Goff L, Pertea G, Kim D, Kelley DR, et al. Differential gene and transcript expression analysis of RNA-seq experiments with TopHat and Cufflinks. Nature Protocols. 2012; 7(3): 562.

35. Haas BJ, Delcher AL, Mount SM, Wortman JR, Smith Jr RK, Hannick LI, et al. Improving the Arabidopsis genome annotation using maximal transcript alignment assemblies. Nucleic Acids Research. 2003; 31(19): 5654-66.

36. Haas BJ, Salzberg SL, Zhu W, Pertea M, Allen JE, Orvis J, et al. Automated

eukaryotic gene structure annotation using EVidenceModeler and the Program to Assemble Spliced Alignments. Genome Biology. 2008; 9(1): R7.

37. Li H, Handsaker B, Wysoker A, Fennell T, Ruan J, Homer N, et al. The sequence alignment/map format and SAMtools. Bioinformatics. 2009; 25(16): 2078-79.

38. Kanehisa M and Goto S. KEGG: kyoto encyclopedia of genes and genomes. Nucleic Acids Research. 2000; 28(1): 27-30.

39. Pruitt KD, Tatusova T, and Maglott DR. NCBI reference sequences (RefSeq): a curated non-redundant sequence database of genomes, transcripts and proteins. Nucleic Acids Research. 2006; 35(suppl_1): D61-D65.

40. Consortium U. UniProt: a hub for protein information. Nucleic Acids Research. 2014: gku989.

41. Hunter S, Apweiler R, Attwood TK, Bairoch A, Bateman A, Binns D, et al. InterPro: the integrative protein signature database. Nucleic Acids Research. 2008; 37(suppl_1): D211-D15.

42. Zdobnov EM and Apweiler R. InterProScan–an integration platform for the signature-recognition methods in InterPro. Bioinformatics. 2001; 17(9): 847-48.

43. Li H, Coghlan A, Ruan J, Coin LJ, Heriche JK, Osmotherly L, et al. TreeFam: a curated database of phylogenetic trees of animal gene families. Nucleic Acids Research. 2006; 34(suppl_1): D572-D80.

44. Ruan J, Li H, Chen Z, Coghlan A, Coin LJ, Guo Y, et al. TreeFam: 2008 Update. Nucleic Acids Research. 2008; 36(Database issue): D735-40.

45. Stamatakis A. RAxML version 8: a tool for phylogenetic analysis and post-

analysis of large phylogenies. Bioinformatics. 2014; 30(9): 1312-13.

46. Sanderson MJ. r8s: inferring absolute rates of molecular evolution and divergence times in the absence of a molecular clock. Bioinformatics. 2003; 19(2): 301-02.

47. Van De Wiel MA, Leday GG, Pardo L, Rue H, Van Der Vaart AW, and Van Wieringen WN. Bayesian analysis of RNA sequencing data by estimating multiple shrinkage priors. Biostatistics. 2013; 14(1): 113-28.

48. De Bie T, Cristianini N, Demuth JP, and Hahn MW. CAFE: a computational tool for the study of gene family evolution. Bioinformatics. 2006; 22(10): 1269-71.

49. ImmunoDB. EM Zdobnov Group. 2008. http://cegg.unige.ch/Insecta/immunodb. Accessed 29 May 2016.

50. Zhao W, Yang PC, Kang L, and Cui F. Different pathogenicities of Rice stripe virus from the insect vector and from viruliferous plants. New Phytologist. 2016; 210(1): 196-207.

51. Wang L, Tang N, Gao XL, Guo DY, Chang ZX, Fu YT, et al. Understanding the immune system architecture and transcriptome responses to southern rice black-streaked dwarf virus in Sogatella furcifera. Scientific reports. 2016; 6.

52. Kim D, Pertea G, Trapnell C, Pimentel H, Kelley R, and Salzberg SL. TopHat2: accurate alignment of transcriptomes in the presence of insertions, deletions and gene fusions. Genome Biology. 2013; 14(4): R36.

53. Anders S, Pyl PT, and Huber W. HTSeq--a Python framework to work with

high-throughput sequencing data. Bioinformatics. 2015; 31(2): 166-69.

54. Zhang F, Li Q, Chen X, Huo Y, Guo H, Song Z, et al. Roles of the Laodelphax striatellus Down syndrome cell adhesion molecule in Rice stripe virus infection of its insect vector. Insect Molecular Biology. 2016; 25(4): 413-21.

Figure legends

Figure 1. Photograph of *Laodelphax striatellus* on a rice plant leaf. Scale bar, 1 mm.

Figure 2. Gene cluster analysis among 22 arthropod species. 1:1:1 and N:N:N represents universal orthologs with single copy or multiple copy number, respectively. Insect, Diptera, Hemiptera, Hymenoptera, Lepidoptera and Coleoptera stand for taxon-specific orthologs, respectively. Other indicates orthlogs that do not belong to any above-mentioned ortholog categories. SD indicates species-specifically duplicated genes. ND indicates genes that cannot be classified into any other categories.

Figure 3. Venn diagram of functional annotation by four databases. NR, non-redundant protein databases; KEGG, Kyoto Encyclopedia of Genes and Genomes.

Figure 4. Phylogenetic analysis of 22 arthropod species. The phylogenetic tree was constructed based on amino acid sequences of 277 single-copy orthologs among 22 arthropod species (*Anopheles gambiae*, *Anoplophora glabripennis*, *Apis mellifera*, *Acyrthosiphon pisum*, *Bombyx mori*, *Bemisia tabaci*, *Cimex lectularius*, *Diaphorina citri*, *Drosophila melanogaster*, *Diuraphis noxia*, *Danaus plexippus*, *Daphnia pulex*, *Locusta migratoria*, *Laodelphax striatellus*, *Nilaparvata lugens*, *Nasonia vitripennis*, *Oncopeltus fasciatus*, *Pediculus humanus*, *Rhodnius prolixus*, *Sogatella furcifera*,

Tribolium castaneum, *Zootermopsis nevadensis*) using maximum likelihood algorithm. The tree was rooted with *D. pulex*.

Table 1. Sequencing data used for genome assembly and annotation.

Category	Accession	Life stage	Insert Size (bp)	Read Length	Reads Number
Survey	SRR5816389	Adult	230	2 x 125	127772669
Assmebly	SRR5830088	Adult	180	2 x 100	123459791
	SRR5816388	Adult	250	2 x 125	137013558
	SRR5816387	Adult	500	2 x 100	141587274
	SRR5816386	Adult	500	2 x 125	30520480
	SRR5816393	Adult	800	2 x 100	153498320
	SRR5816392	Adult	1.4-1.6 K	2 x 125	40251413
	SRR5816391	Adult	2.6-2.8 K	2 x 125	36559438
	SRR5816390	Adult	5-5.6 K	2 x 125	26684783
	SRR5816385	Adult	5.6-6.5 K	2 x 125	23069935
	SRR5816384	Adult	9-11 K	2 x 125	24285333
	SRR5816377	Adult	11-13 K	2 x 125	23396366
	SRR5816376	Adult	13-15 K	2 x 125	30547732
	SRR5816379	Adult	15-18 K	2 x 125	25926919
	SRR5816378	Adult	18-24 K	2 x 125	26325395
	SRR5817574	Adult	-	8559	99701
	SRR5817559	Adult	-	8947	77038
	SRR5817582	Adult	-	8474	104288
	SRR5817569	Adult	-	8518	114320
	SRR5817560	Adult	-	9202	80599
	SRR5817562	Adult	-	9211	100089
	SRR5817573	Adult	-	8610	102997
	SRR5817558	Adult	-	9007	86083
	SRR5817581	Adult	-	8452	89374
	SRR5817570	Adult	-	8419	101715
	SRR5817550	Adult	-	9192	82657
	SRR5817576	Adult	-	8597	105080
	SRR5817553	Adult	-	8586	77467
	SRR5817557	Adult	-	8821	75712
	SRR5817567	Adult	-	8363	106634
	SRR5817575	Adult	-	8620	105795
	SRR5817552	Adult	-	8985	66096
	SRR5817556	Adult	-	8573	83500
	SRR5817568	Adult	-	8357	104295
	SRR5817578	Adult	-	8528	108299
	SRR5817565	Adult	-	8728	69694
	SRR5817555	Adult	-	8480	86385
	SRR5817571	Adult	-	8437	106314
	SRR5817577	Adult	-	8686	106337
	SRR5817566	Adult	-	8890	52889

	SRR5817554	Adult	-	8648	85970
	SRR5817572	Adult	-	8437	101258
	SRR5817580	Adult	-	8490	104459
	SRR5817563	Adult	-	8954	91218
	SRR5817561	Adult	-	8724	84033
	SRR5817579	Adult	-	8776	107138
	SRR5817564	Adult	-	9054	68294
	SRR5817551	Adult	-	8508	88776
Annotation	SRR5816381	Larva	250-300	2 x 150	23733333
	SRR5816380	Adult	250-300	2 x 150	24933333
	SRR5816383	Egg	250-300	2 x 150	24633333
	SRR5816382	Fat body	250-300	2 x 150	31300000
	SRR5816375	Brain	250-300	2 x 150	40333333
	SRR5816374	Gonad	250-300	2 x 150	33300000
	SRR5816394	Tentacle	250-300	2 x 150	24966666

Note: Survey library in the Category column was used to estimate the genome size of Laodelphax striatellus. Libraries of insert size >1 Kb were mate-paired. For gene annotation, data from two previously sequenced tissues were used under accession SRR1619428 for salivary gland and SRR1617617 for alimentary canal.

Table 2. Statistics comparison of genome assembly and annotation among three planthoppers.

Category	*Laodelphax striatellus*		*Nilaparvata lugens*[a]		*Sogatella furcifera*[b]	
	Contig	Scaffold	Contig	Scaffold	Contig	Scaffold
Total size (Mb)	530.2	541.0	993.8	1140.8	673.9	720.7
Total number	48574	38193	80046	46558	50020	20450
Maximum length (Kb)	1990	10350	230	2254	800	12789
N50 length (Kb)	118	1085	24	357	71	1185
GC content (%)		34.5		34.6		31.6
TE proportion (%)		23.0		38.9		39.7
BUSCO evaluation (%)		92		81		92
Gene number		17736		27571		21254
Average gene length (bp)		14342		11216		12597
Average CDS length (bp)		1289		1135		1526
Average exon per gene		6		4		6
Average exon length (bp)		213		264		240
Average intron length (bp)		2587		3062		2064

Note: TE, transposable element; BUSCO, benchmarking universal single copy ortholog; CDS, coding sequence; Gene number means number of protein-coding genes.

[a]From the published *Nilaparvata lugens* genome [8].

[b]From the published *Sogatella furcifera* genome [9].

Table 3. Comparison of transposable element (TE) contents of the three planthoppers.

Class	*Laodelphax striatellus*						*Nilaparvata lugens*		*Sogatella furcifera*	
	De novo + Repbase		TE Proteins		Combined TEs		Combined TEs		Combined TEs	
	Length (bp)	% of genome	Length (bp)	% of genome	Length (bp)	% of genome	Length (bp)	% of genome	Length (bp)	% of genome
DNA	24818676	4.59	2550902	0.47	26592872	4.92	162024958	14.20	126002323	17.33
LINE	24160245	4.47	4889094	0.90	27124925	5.01	182652892	16.00	69257982	9.52
LTR	7122249	1.32	0	0.00	7122249	1.32	168492299	14.80	31286552	4.30
SINE	22739683	4.20	743909	0.14	23044510	4.26	8272412	0.70	10730722	1.48
Other	0	0.00	0	0.00	0	0.00	41262	0.00	23167338	3.18
Unknown	27609625	5.10	0	0.00	27609625	5.10	21890733	1.90	28395639	3.90
Total	119645576	22.12	8177428	1.51	124360921	22.99	443765874	38.90	288840556	39.73

Note: De novo + Repbase refers to TE integrated between de novo and Repbase prediction. TE proteins refers to TE identified by RepeatProteinMask. Combined TEs refers to TE combined two results above. DNA, DNA transposon; LINE, long interspersed nuclear element; LTR, long terminal repeat; SINE, short interspersed nuclear element. Other means TE that can be classified but doesn’t belong given classes. Unknown means TE that can’t be classified.

Additional file 1

Table S1. Base composition of the *Laodelphax striatellus* genome assembly.

Table S2. Summary of reads mapping to the genome assembly of *Laodelphax striatellus*.

Table S3. Transcript-based evaluation of the genome assembly of *Laodelphax striatellus*.

Table S4. Statistics of nine transcriptomic reads mapped to different genomic regions.

Table S5. Genome completeness assessment using benchmarking universal single copy orthologs in five insects.

Table S6. Repetitive elements predicted by different programs.

Table S7. Sources of genome data of 22 arthropod species.

Table S8. Gene models predicted by different methods.

Table S9. Statistical comparison of gene sets of *Laodelphax striatellus* and 9 other arthropod species.

Table S10. Expanded gene families in the three planthoppers.

Table S11. Chemoreception related genes in the three planthoppers.

Table S12. Detoxification related genes in the three planthoppers.

Table S13. Immune genes in the three planthoppers.

Table S14. Sources of gene annotation files for the three planthoppers.

Table S15. Shared Gene Ontology terms for differentially expressed genes in the three planthoppers responding to plant viruses.

Table S16. Commonly regulated genes with similar functions in the three planthoppers responding to plant viruses.

Table S17. Homologous genes in the three planthoppers responding to plant viruses.

Additional file 2

Figure S1. *Laodelphax striatellus* genome size estimation by flow cytometry and k-mer analyses. (A), (B) and (C) showed fluorescence peaks for *Drosophila melanogaster, Gallus gallus* and *L. striatellus*, respectively. The genome sizes of *D. melanogaster* and *G. gallus* were 0.18 pg and 1.25 pg, respectively. The genome size of *L. striatellus* was calculated to be 0.60 pg. (D) illustrated the depth distribution of k-mers (k = 17).

Figure S2. *Laodelphax striatellus* chromosomes dyed with Hoechst 33258. (A) haploid chromosomes. (B) diploid chromosomes.

Figure S3. Sequencing depth distribution. The x-axis shows sequencing depth and the y-axis shows fraction of bases with certain sequencing depth.

Figure S4. Summary of gene structures of *Laodelphax striatellus* and eight other species used for gene annotation.

Figure S5. Benchmarking universal single copy orthologs (BUSCO) assessment of the *Laodelphax striatellus* gene set. The completeness of the gene set was assessed with two BUSCO Ver. 2 datasets (arthropoda and eukaryote). The recovered matches are classified as ‘complete’ if their lengths are within the expectation of the BUSCO

profile match lengths. If these are found only once they are classified as ‘complete single’ and other ‘complete’ matches are classified as ‘complete duplicated’. The matches that are only partially recovered are classified as ‘fragmented’, and BUSCO groups for which there are no matches that pass the tests of orthology are classified as ‘missing’. For each species, the right bar shows the arthropoda results and the left bar shows the eukaryote results. Aga, *Anopheles gambiae*; Agl, *Anoplophora glabripennis*; Ame, *Apis mellifera*; Api, *Acyrthosiphon pisum*; Bmo, *Bombyx mori*; Bta, *Bemisia tabaci*; Cle, *Cimex lectularius*; Dci, *Diaphorina citri*; Dme, *Drosophila melanogaster*; Dno, *Diuraphis noxia*; Dpl, *Danaus plexippus*; Dpu, *Daphnia pulex*; Lmi, *Locusta migratoria*; Lst, *Laodelphax striatellus*; Nlu, *Nilaparvata lugens*; Nvi, *Nasonia vitripennis*; Ofa, *Oncopeltus fasciatus*; Phu, *Pediculus humanus*; Rpr, *Rhodnius prolixus*; Sfu, *Sogatella furcifera*; Tca, *Tribolium castaneum*; Zne, *Zootermopsis nevadensis*.

Figure S6. Determination of genomic heterozygosity. (A) Density distribution of heterozygous rates. (B) Frequency distribution of read coverage of both high and low heterozygosity. All heterozygosity rates were ranked and the top 20% was chosen as high heterozygosity (high_het in the legend) and the left as low heterozygosity (low_het in the legend).

Figure S7. Divergence times estimation of 22 arthropod species. The number on each node stands for the divergence time from the present (million years ago, Mya) with 95% confidence interval values noted in brackets. Four calibration time were used in the estimation: *D. pulex-D. melanogaster* divergence (445~530 Mya), *N. vitripennis*-

D. melanogaster divergence (279~306 Mya), *A. gambiae-D. melanogaster* divergence (235~269 Mya) and *A. mellifera-N. vitripennis* divergence (175~215 Mya).

Figure S8. Gene family expansion and contraction in the three planthoppers. *R. prolixus* was used as outgroup to construct the phylogenetic tree and infer expanded/contracted gene families by CAFÉ. A conditional *P*-value was calculated for each gene family and families with *P*-value < 0.05 were considered as significantly expanded (green) or contracted (red).

Figure 1 Click here to download Figure Figure 1.tif

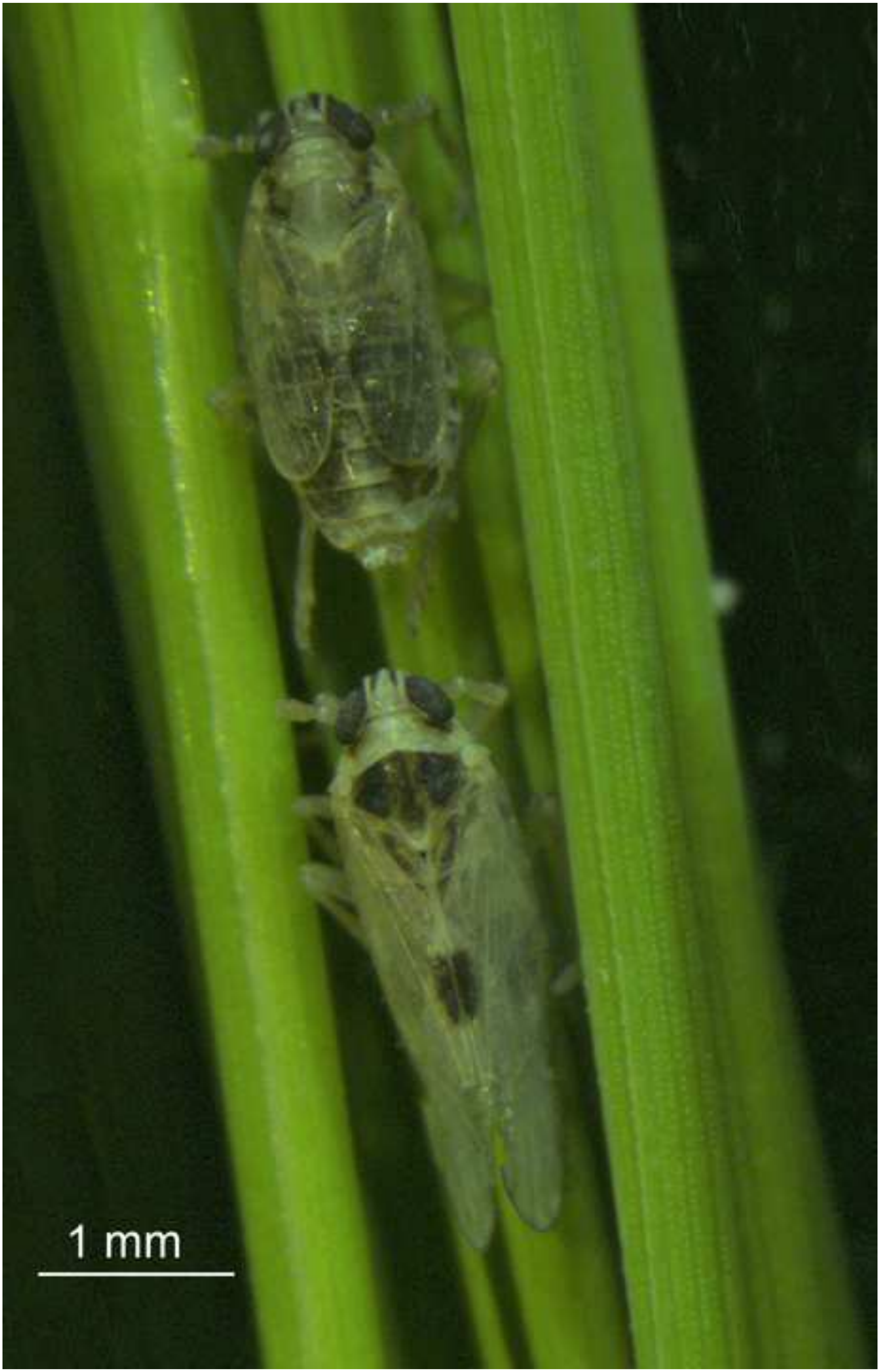

Figure 2

Click here to download Figure Figure 2.pdf

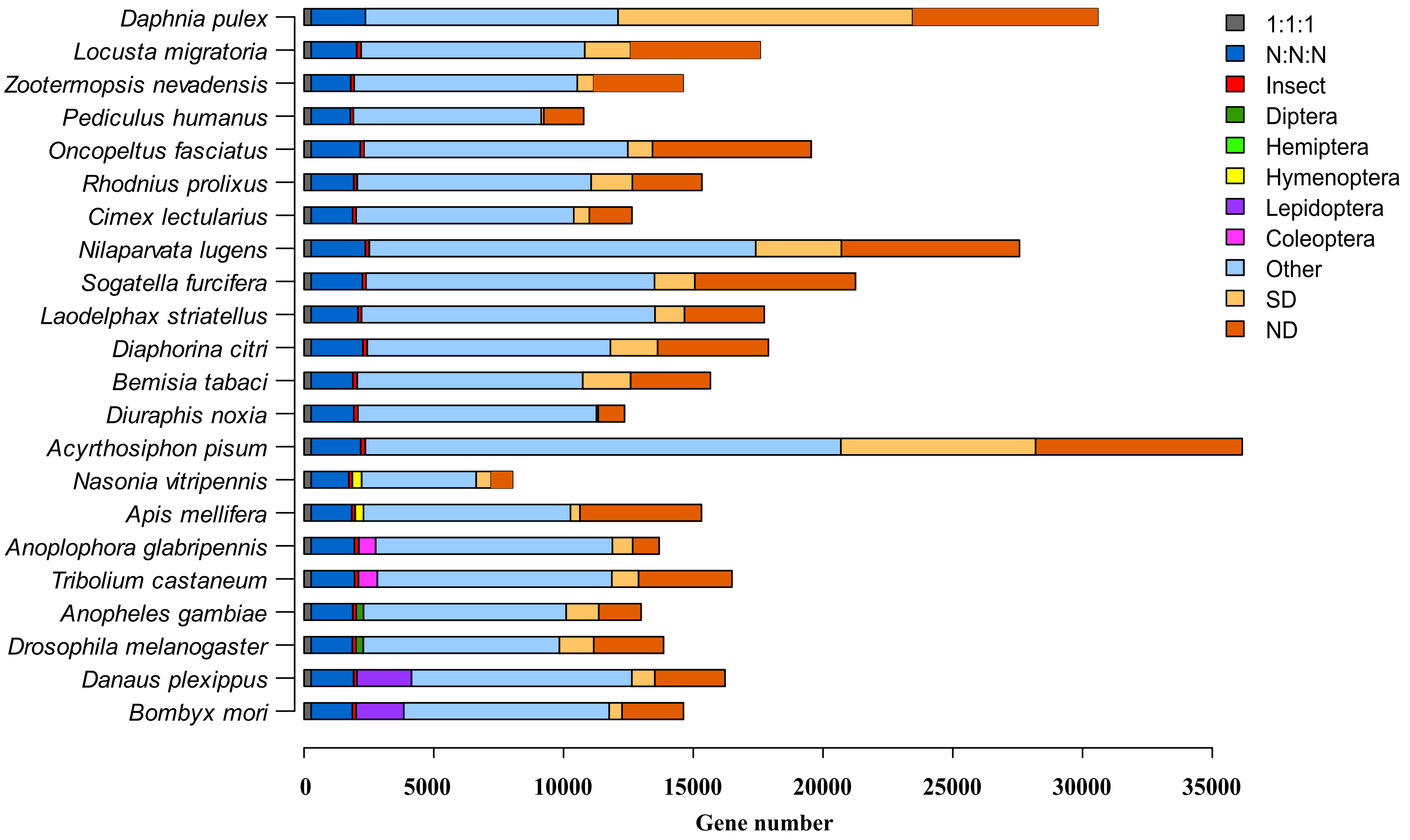

Figure 3

Click here to download Figure Figure 3.pdf

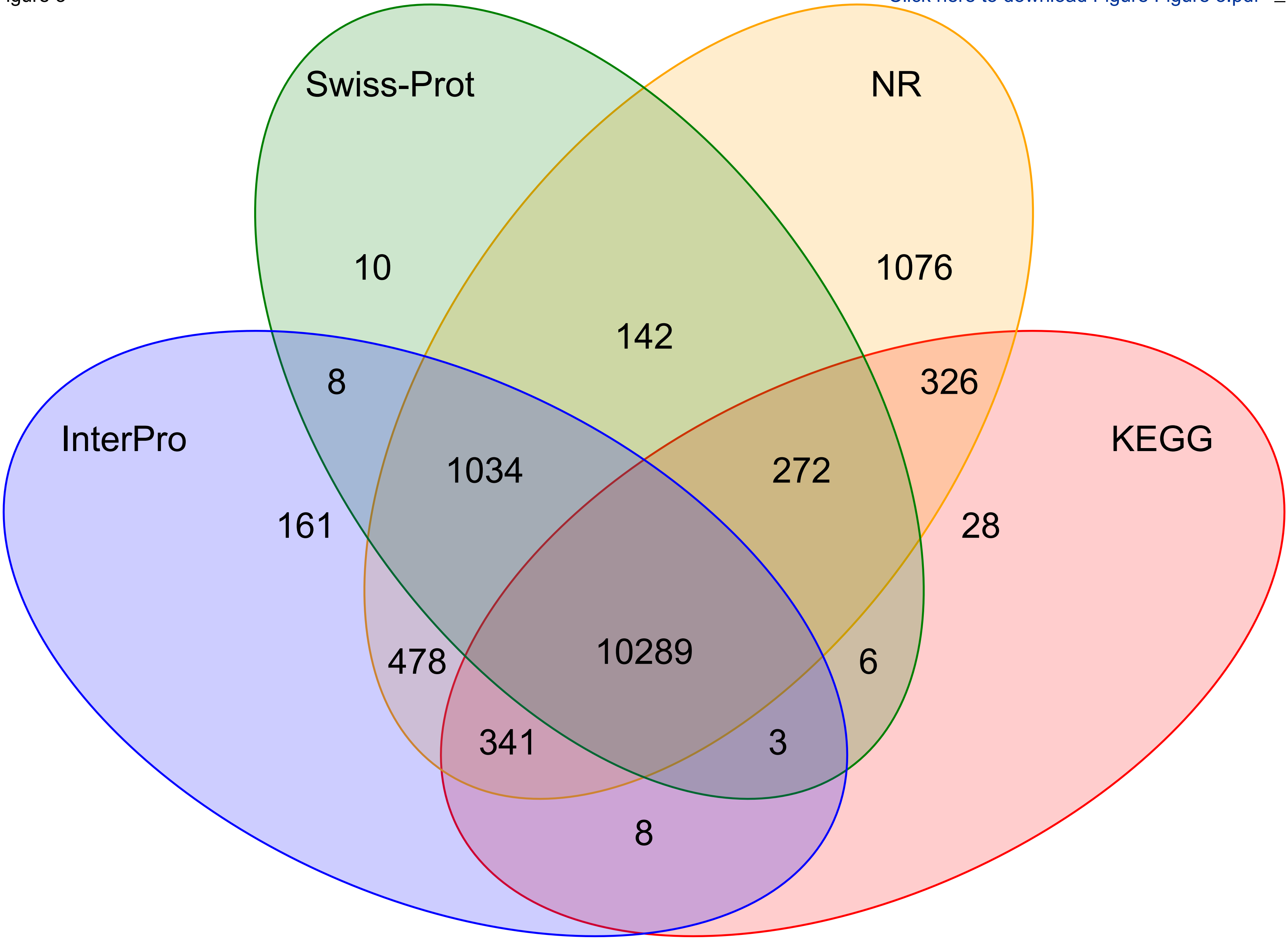

Figure 4

Click here to download Figure Figure 4.jpg

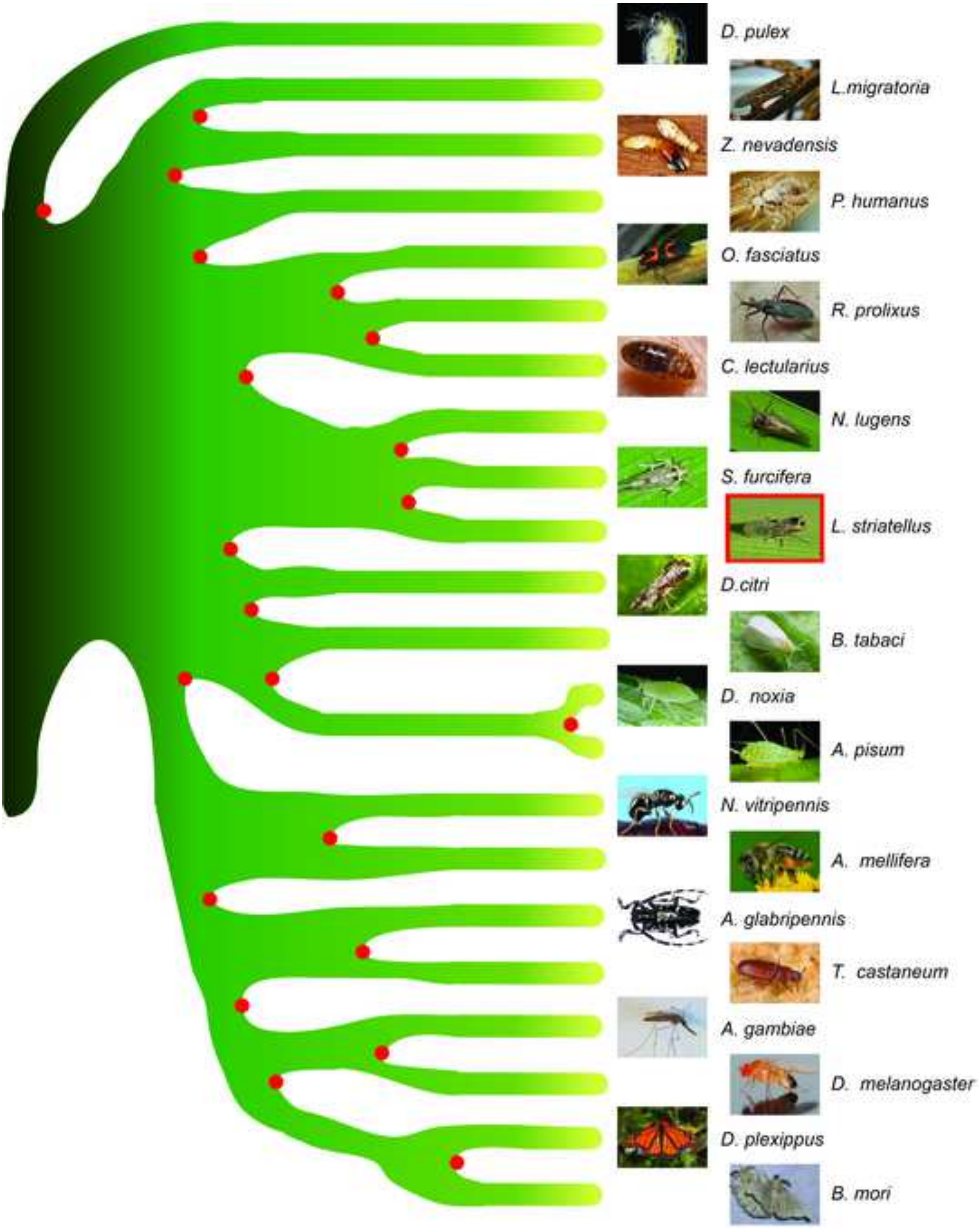

Additional file 1

Click here to access/download
Supplementary Material
Additional file 1.pdf

Additional file 2

Click here to access/download
Supplementary Material
Additional file 2.pdf
