# Supplementary material for: Genome sequence of the small brown planthopper, Laodelphax striatellus
Source: Gigascience. 2017 Nov 10;6(12):1–12. doi: 10.1093/gigascience/gix109 (PMC5740986; doi:10.1093/gigascience/gix109)
Supplement: Additional Files [file gix109_supp.zip › Additional file 1.pdf]

Table S1. Base composition of the *Laodelphax striatellus* genome assembly.

Base	Number (bp)	% of genome
A	173,426,040	32.06
T	173,671,001	32.10
C	91,600,949	16.93
G	91,505,534	16.92
N	10,761,718	1.99
Total	540,965,242	100
GC	183,106,483	34.54

Note: GC in the Base column means GC content of the genome assembly without N.

Table S2. Summary of reads mapping to the genome assembly of *Laodelphax striatellus*.

Category	Percentage (%)
Coverage	96.83
Coverage at least 4X	96.29
Coverage at least 10X	95.77
Coverage at least 20X	95.08

Note: Coverage, percentage of genome covered by reads; Coverage at least NX, percentage of genome covered at least NX.

Table S3. Transcript-based evaluation of the genome assembly of *Laodelphax striatellus*.

Dataset	Number	Total length (bp)	Covered by assembly (%)	With >90% sequence in one scaffold		With >50% sequence in one scaffold	
				Number	Percentage (%)	Number	Percentage (%)
>0 bp	9,796	11,052,346	99.87	8,847	90.31	9,685	98.87
>200 bp	9,796	11,052,346	99.87	8,847	90.31	9,685	98.87
>500 bp	6,359	9,936,718	99.98	5,699	89.62	6,287	98.87
>1 Kb	3,978	8,224,163	99.98	3,510	88.24	3,923	98.62
>2 Kb	1,517	4,712,766	100.00	1,311	86.42	1,489	98.15

Table S4. Statistics of nine transcriptomic reads mapped to different genomic regions.

Sample accession	Coverage (%)		
	Exon	Intron	Intergenic
SRR5816381	79.61	6.80	13.59
SRR5816380	79.87	6.03	14.10
SRR5816383	77.97	5.35	16.68
SRR5816394	84.60	5.59	9.81
SRR5816375	85.49	4.43	10.08
SRR5816382	83.28	6.14	10.58
SRR5816374	87.10	5.07	7.84
SRR1617617	80.86	7.19	11.96
SRR1619428	94.10	2.03	3.87

Note: Details about the nine transcriptomes are listed in Table 1. Genomic regions are divided into exon, intron and intergenic region.

Table S5. Genome completeness assessment using benchmarking universal single copy orthologs in five insects

Species	Complete Single-copy	Complete Duplicated	Fragmented	Missing	Total	Genome
Laodelphax striatellus	2020	106	450	205	2675	C: 75% [D: 3.9%], F: 16%, M: 7.6%, N: 2675
Nilaparvata lugens	1606	194	692	377	2675	C: 60% [D: 7.2%], F: 25%, M: 14%, N: 2675
Rhodnius prolixus	1983	65	522	170	2675	C: 74% [D: 2.4%], F: 19%, M: 6.3%, N: 2675
Acyrthosiphon pisum	1839	156	464	372	2675	C: 68% [D: 5.8%], F: 17%, M: 13%, N: 2675
Sogatella furcifera	2065	197	428	182	2675	C: 77% [D: 7.3%], F: 16%, M: 6.8%, N: 2675

Note: C, D, F and M in the Genome column stand for complete, complete but duplicated, fragmented, missing orthologs in the genome, respectively. N represents the number of all BUSCO arthropoda (Ver. 1) genes.

Table S6. Repetitive elements predicted by different programs.

Type	Size (bp)	% of genome
Tandem Repeat Finder	27,291,295	5.05
RepeatMasker	119,645,576	22.12
RepeatProteinMask	8,177,428	1.51
Total	139,107,266	25.71

Table S7. Sources of genome data of 22 arthropod species.

Species	Abbreviation	Downloading site
Anopheles gambiae	Aga	ftp://ftp.ensemblgenomes.org/pub/metazoa/release-34/fasta/anopheles_gambiae
Anoplophora glabripennis	Agl	ftp://ftp.ncbi.nlm.nih.gov/genomes/all/GCF/000/390/285/GCF_000390285.1_Agla_1.0
Apis mellifera	Ame	http://hymenopteragenome.org/beebase/?q=consortium_datasets
Acyrthosiphon pisum	Api	ftp://ftp.ensemblgenomes.org/pub/metazoa/release-34/fasta/acyrthosiphon_pisum
Bombyx mori	Bmo	ftp://ftp.ensemblgenomes.org/pub/metazoa/release-34/fasta/bombyx_mori
Bemisia tabaci	Bta	ftp://whiteflygenomics.org/pub/MEAM1/v1.1/
Cimex lectularius	Cle	ftp://ftp.ncbi.nlm.nih.gov/genomes/refseq/invertebrate/Cimex_lectularius/latest_assembly_versions/GCF_000648675.1_Clec_1.0/
Diaphorina citri	Dci	ftp://ftp.ncbi.nlm.nih.gov/genomes/all/GCF/000/475/195/GCF_000475195.1_Diaci_psyllid_genome_assembly_version_1.1
Drosophila melanogaster	Dme	ftp://ftp.ensemblgenomes.org/pub/metazoa/release-32/fasta/drosophila_melanogaster/
Diuraphis noxia	Dno	ftp://ftp.ncbi.nlm.nih.gov/genomes/all/GCF/001/186/385/GCF_001186385.1_Dnoxia_1.0
Danaus plexippus	Dpl	ftp://ftp.ensemblgenomes.org/pub/metazoa/release-34/fasta/danaus_plexippus
Daphnia pulex	Dpu	ftp://ftp.ncbi.nlm.nih.gov/genomes/all/GCA/000/187/875/GCA_000187875.1_V1.0
Locusta migratoria	Lmi	unpublished data
Laodelphax striatellus	Lst	This study
Nilaparvata lugens	Nlu	http://gigadb.org/dataset/100139
Nasonia vitripennis	Nvi	ftp://ftp.ensemblgenomes.org/pub/metazoa/release-34/fasta/nasonia_vitripennis
Oncopeltus fasciatus	Ofa	https://i5k.nal.usda.gov/data/Arthropoda/oncfas-%28Oncopeltus_fasciatus%29/Current%20Genome%20Assembly/2.Official%20or%20Primary%20Gene%20Set/OGS_v1_1/
Pediculus humanus	Phu	ftp://ftp.ensemblgenomes.org/pub/metazoa/release-34/fasta/pediculus_humanus
Rhodnius prolixus	Rpr	ftp://ftp.ensemblgenomes.org/pub/metazoa/release-34/fasta/rhodnius_prolixus
Sogatella furcifera	Sfu	ftp://climb.genomics.cn/pub/10.5524/100001_101000/100255/
Tribolium castaneum	Tca	ftp://ftp.ensemblgenomes.org/pub/metazoa/release-34/fasta/tribolium_castaneum

Zootermopsis nevadensis	Zne	ftp://ftp.ncbi.nlm.nih.gov/genomes/all/GCA/000/696/155/GCA_000696155.1_ZooNev1.0

Table S8. Gene models predicted by different methods.

Gene set		Number	Average gene length (bp)	Average CDS length (bp)	Average exons per gene	Average exon length (bp)	Average intron length (bp)
De novo	Augustus	29,929	7,369.78	891.76	4.21	212.01	2,020.43
	GlimmerHMM	57,243	7,503.29	545.69	3.31	165.06	3,017.23
	SNAP	61,952	11,500.85	686.04	4.34	158.03	3,236.68
	GeneID	52,468	7,263.59	284.66	2.68	106.05	4,144.12
	Genscan	32,610	9,916.76	894.86	4.22	212.24	2,805.13
**Homology**	*Bombyx mori*	11,029	5,350.02	834.93	3.48	240.23	1,823.86
	Rhodnius prolixus	12,725	5,984.01	892.72	3.84	232.58	1,793.80
	Acyrthosiphon pisum	11,625	6,158.68	915.23	3.73	245.07	1,917.49
	Daphnia pulex	8,951	7,451.36	935.82	4.29	218.21	1,81.27
	Drosophila melanogaster	6,710	10,033.21	1,155.99	5.36	215.73	2,036.73
	Nasonia vitripennis	9,412	7,652.37	1,040.11	4.34	239.77	1,980.98
	Nilaparvata lugens	18,824	5,629.43	990.04	3.85	257.01	1,626.65
	Pediculus humanus	9,133	9,006.65	1,122.29	5.05	222.45	1,949.05
**RNA-seq**	Cufflinks	43,650	23,904.39	2,859.85	7.18	398.54	3,407.58
	PASA	29,350	24,224.51	1,543.74	7.65	201.92	3,413.05
**EVM**		26,667	10,058.86	967.28	4.60	210.5	2,528.87
**Final set**		17,736	14,342.38	1,288.70	6.05	213.16	2,587.13

Table S9. Statistical comparison of gene sets of *Laodelphax striatellus* and 9 other arthropod species.

Species	Number	Average gene length (bp)	Average CDS length (bp)	Average exons per gene	Average exon length (bp)	Average intron length (bp)
Laodelphax striatellus	17,736	14,342.38	1,288.70	6.05	213.16	2,587.13
Nilaparvata lugens	27,571	11,216.07	1,135.02	4.29	264.43	3,061.91
Sogatella furcifera	21,254	12,597.22	1,525.90	6.36	239.82	2,064.50
Acyrthosiphon pisum	36,195	6,050.76	1,157.81	4.54	254.86	1,381.05
Pediculus humanus	10,786	3,136.00	1,543.88	6.42	240.38	293.61
Nasonia vitripennis	8,037	6,578.55	1,622.12	6.34	255.82	928.01
Daphnia pulex	30,590	2,019.05	983.70	4.65	211.66	283.85
Drosophila melanogaster	13,913	4,487.84	1,610.58	3.96	406.64	971.82
Bombyx mori	14,623	6,043.48	1,212.73	5.46	222.27	1,084.10
Rhodnius prolixus	15,438	7,353.42	1,059.56	5.77	183.50	1,318.33

Note: CDS, coding sequence.

Table S10. Expanded gene families in the three planthoppers.

Species	Gene family	*Laodelphax striatellus*	*Sogatella furcifera*	*Nilaparvata lugens*	*P*-value
Laodelphax striatellus	odorant receptor	6	0	0	0.048
	cytochrome P450	5	2	1	0.045
	serine protease	2	0	0	0.048
	chemosensory protein	5	1	3	>0.05
	odorant binding protein	4	1	2	>0.05
	carboxylesterase	7	4	4	>0.05
	ATP-binding cassette transporter	9	7	6	>0.05
Sogatella furcifera	acyl-CoA synthetase	7	20	4	0.000
	fatty acyl-CoA reductase	2	6	1	0.000
	acyl-CoA-binding protein	0	2	0	0.048
	acyl-coenzyme A thioesterase	0	2	0	0.048
	glyceraldehyde-3-phosphate dehydrogenase	3	5	1	>0.05
	D-beta-hydroxybutyrate dehydrogenase	1	3	1	>0.05
	ADP/ATP translocase	2	3	1	>0.05
	acyl-CoA transporter	1	2	1	>0.05
	ATP synthase	2	4	2	>0.05
Nilaparvata lugens	Delta(3,5)-Delta(2,4)-dienoyl-CoA isomerase	2	1	4	>0.05
	ATP-citrate synthase	1	1	2	>0.05
	malonyl-CoA decarboxylase	1	1	2	>0.05
	NADH dehydrogenase (ubiquinone) 1α subcomplex subunit 7 and subunit 8	2	1	4	>0.05

	acyl-CoA synthetase	1	1	2	>0.05
	ATP synthase	2	2	4	>0.05
	enoyl-CoA delta isomerase	1	1	2	>0.05

Note: The three columns between Gene family column and *P*-value column showed the number of certain gene family in the three planthoppers.

Table S11. Chemoreception related genes in the three planthoppers.

Species	OR	IR	GR	OBP	CSP
Laodelphax striatellus	106	38	12	16	31
Sogatella furcifera	39	22	5	14	24
Nilaparvata lugens	44	44	4	10	27

Note: Chemoreception related genes include odorant receptors (ORs), gustatory receptors (GRs), iontropic receptors (IRs), odorant binding proteins (OBPs) and chemosensory proteins (CSPs).

Table S12. Detoxification related genes in the three planthoppers.

Species	ABC transporter	CCE	CYP	UGT	GST
Laodelphax striatellus	73	54	76	26	29
Sogatella furcifera	74	41	66	19	28
Nilaparvata lugens	87	61	79	19	27

Note: Detoxification related genes include UDP-glycosyltransferases (UGTs), glutathione-S-transferases (GSTs), carboxyl/cholinesterase (CCEs), ATP-binding cassette transporters (ABC transporters), and cytochrome P450s (CYPs).

Table S13. Immune genes in the three planthoppers.

Immune pathway	Lst	Nlu	Sfu
Anti-Microbial Peptides	1	1	1
Autophagy Genes	74	57	58
1,3-beta-D Glucan Binding Proteins	8	4	2
Caspases	2	2	3
Catalases	1	1	1
Clip-Domain Serine Proteases	53	40	43
C-Type Lectins	7	11	14
Fibrinogen-Related Proteins	0	3	1
Galactoside-Binding Lectins	2	2	1
Inhibitors of Apoptosis	4	6	3
IMD Pathway Members	8	4	7
Signal Transduction	3	3	3
Lysozymes	1	1	1
MD2-Like Receptors	5	2	4
Peptidoglycan Recognition Proteins	2	0	0
Peroxidases	19	19	19
Prophenoloxidases	4	5	4
Relish-like Proteins	1	0	1
Scavenger Receptors	17	13	17
Superoxide Dismutases	5	3	2
Spaetzle-like Proteins	3	3	2
Serine Protease Inhibitors	7	7	7
Small RNA Regulatory Pathway Members	86	74	66
Thioester-Containing Proteins	4	3	3
Toll-like Receptors	6	16	14
Toll Pathway Members	7	9	3
Total	330	289	280

Note: Lst, *Laodelphax striatellus*; Nlu, *Nilaparvata lugens*; Sfu, *Sogatella furcifera*.

Table S14. Sources of gene annotation files for the three planthoppers.

Species	Websites
Laodelphax striatellus	This study
Nilaparvata lugens	http://gigadb.org/dataset/100255
Sogatella furcifera	http://gigadb.org/dataset/100139

Table S15. Shared Gene Ontology terms for differentially expressed genes in the three planthoppers responding to plant viruses.

Regulation	GO ID	Category	Description
Up-regulated	GO:0000166	MF	nucleotide binding
	GO:0003676	MF	nucleic acid binding
	GO:0003677	MF	DNA binding
	GO:0003723	MF	RNA binding
	GO:0004672	MF	protein kinase activity
	GO:0005506	MF	iron ion binding
	GO:0005515	MF	protein binding
	GO:0005524	MF	ATP binding
	GO:0005525	MF	GTP binding
	GO:0008270	MF	zinc ion binding
	GO:0020037	MF	heme binding
	GO:0046872	MF	metal ion binding
	GO:0005634	CC	nucleus
	GO:0006355	BP	regulation of transcription, DNA-templated
	GO:0006468	BP	protein phosphorylation
	GO:0055114	BP	oxidation-reduction process
Down-regulated	GO:0004181	MF	metallocarboxypeptidase activity
	GO:0004252	MF	serine-type endopeptidase activity
	GO:0004568	MF	chitinase activity
	GO:0005488	MF	binding
	GO:0005506	MF	iron ion binding
	GO:0005509	MF	calcium ion binding
	GO:0005515	MF	protein binding
	GO:0008270	MF	zinc ion binding
	GO:0016491	MF	oxidoreductase activity
	GO:0016705	MF	oxidoreductase activity, acting on paired donors, with incorporation or reduction of molecular oxygen
	GO:0020037	MF	heme binding
	GO:0042302	MF	structural constituent of cuticle
	GO:0052689	MF	carboxylic ester hydrolase activity
	GO:0005576	CC	extracellular region
	GO:0016020	CC	membrane
	GO:0005975	BP	carbohydrate metabolic process
	GO:0006032	BP	chitin catabolic process
	GO:0006508	BP	proteolysis
	GO:0008152	BP	metabolic process
	GO:0055114	BP	oxidation-reduction process

Note: We referred genes with higher expressions in the viruliferous group as up-regulated genes and lower as down-regulated. MF, molecular function; BP, biological process; CC, cellular

component.

Table S16. Commonly regulated genes with similar functions in the three planthoppers responding to plant viruses.

Sequence name	Species	Hit description	E-Value	Similarity (%)
**Up-regulated**				
NLU002101.1	*N. lugens*	PREDICTED: gastrula zinc finger protein XlCGF71.1-like isoform X1 [Parasteatoda tepidariorum]	1.10E-30	51.93133
evm.model.Contig498.35	*L. striatellus*	PREDICTED: oocyte zinc finger protein XlCOF6-like [Austrofundulus limnaeus]	1.43E-44	41.33333
evm.model.Contig32.77	*L. striatellus*	zinc finger protein [Nilaparvata lugens]	0	74.21384
Sfur-30.59	*S. furcifera*	PREDICTED: zinc finger MYM-type protein 1-like [Acyrthosiphon pisum]	1.22E-138	56.96552
Sfur-3687.1	*S. furcifera*	Zinc finger MYM-type protein 3 [Zootermopsis nevadensis]	2.28E-07	40.36458
Sfur-467.6	*S. furcifera*	PREDICTED: zinc finger matrin-type protein 2 isoform X1 [Halyomorpha halys]	1.22E-90	88.61386
Sfur-539.11	*S. furcifera*	PREDICTED: gastrula zinc finger protein XlCGF57.1-like [Cimex lectularius]	1.98E-106	67.96117
Sfur-597.15	*S. furcifera*	PREDICTED: zinc finger protein 512B-like, partial [Diaphorina citri]	8.90E-21	58.41121
Sfur-80.12	*S. furcifera*	PREDICTED: gastrula zinc finger protein XlCGF57.1-like [Nothobranchius furzeri]	3.31E-133	65.64103
**Down-regulated**				
NLU020784.1	*N. lugens*	chitinase [Nilaparvata lugens]	0	99.51846
NLU015505.1	*N. lugens*	PREDICTED: probable endochitinase, partial [Diaphorina citri]	2.81E-22	47.05882
NLU012683.1	*N. lugens*	cytochrome P450 CYP4C62v2 [Nilaparvata lugens]	0	100
NLU012996.2	*N. lugens*	trypsin-2 [Nilaparvata lugens]	1.73E-160	99.55157
NLU001881.1	*N. lugens*	trypsin-4 [Nilaparvata lugens]	0	99.30314
evm.model.Contig371.8	*L. striatellus*	chitinase [Nilaparvata lugens]	0	87.08709
evm.model.Contig699.10	*L. striatellus*	chitinase, partial [Nilaparvata lugens]	6.34E-44	86.86869

evm.model.Contig30.151.1	*L. striatellus*	PREDICTED: probable chitinase 3 [Cimex lectularius]	1.7E-143	85.27132
evm.model.Contig136.3	*L. striatellus*	CYP4DJ2 [Nilaparvata lugens]	7.5E-105	95.37572
evm.model.Contig16750.1	*L. striatellus*	PREDICTED: probable endochitinase [Cimex lectularius]	1.44E-31	89.23077
evm.model.Contig45.74	*L. striatellus*	trypsin-12 [Nilaparvata lugens]	0	94.19795
evm.model.Contig8.104	*L. striatellus*	trypsin-19 [Nilaparvata lugens]	0	93.20388
evm.model.Contig100.176	*L. striatellus*	trypsin-2 [Nilaparvata lugens] lcl|AID60340.1 trypsin-6 [Nilaparvata lugens]	0	91.89944
evm.model.Contig100.177	*L. striatellus*	trypsin-5 [Nilaparvata lugens]	0	98.85057
evm.model.Contig480.15	*L. striatellus*	trypsin-7 [Nilaparvata lugens]	0	97.96438
Sfur-134.47	*S. furcifera*	chitinase [Nilaparvata lugens]	3.08E-84	65.4902
Sfur-534.20	*S. furcifera*	chitinase [Nilaparvata lugens]	0	83.33333
Sfur-180.1	*S. furcifera*	cytochrome P450 CYP4C62 [Laodelphax striatella]	0	98.95397
Sfur-619.9	*S. furcifera*	trypsin-22 [Nilaparvata lugens]	1.92E-107	92.85714
Sfur-1563.2	*S. furcifera*	trypsin-3, partial [Nilaparvata lugens]	4.42E-21	96.07843

Note: Sequences with NLU, evm.model.Contig, Sfur prefixes are genes from *Nilaparvata lugens*, *Laodelphax striatellus*, *Sogatella furcifera*, respectively. Up- or down- regulated in the Category column stand for up-/down-regulated genes in the three planthoppers.

Table S17. Homologous genes in the three planthoppers responding to plant viruses.

Category	*Nilaparvata lugens*		*Laodelphax striatellus*			*Sogatella furcifera*		
	Gene ID	Gene length (AA)	Gene ID	Gene Length (AA)	Identity (%)	Gene ID	Gene Length (AA)	Identity (%)
Up-regulated	NLU009517.1	137	evm.model.Contig448.15	139	80	Sfur-169.44	461	78
	NLU027771.1	268			88			78
Down-regulated	NLU005408.1	120	evm.model.Contig350.70	472	72	Sfur-18.59	230	61
	NLU016497.1	148	evm.model.Contig337.14	252	71	Sfur-231.24	217	70

Note: AA is short for amino acids. Sequences with NLU, evm.model.Contig, Sfur prefixes are genes from *N. lugens*, *L. striatellus*, *S. furcifera*, respectively. Up- or down- regulated in the Category column stand for up-/down-regulated genes in the three planthoppers. Genes with identity > 60% and coverage > 50% are considered as genes with sequence similarity.
