# Supplementary material for: Genome sequence of the small brown planthopper, Laodelphax striatellus
Source: Gigascience. 2017 Nov 10;6(12):1–12. doi: 10.1093/gigascience/gix109 (PMC5740986; doi:10.1093/gigascience/gix109)
Supplement: Additional Files [file gix109_supp.zip › Additional file 2.pdf]

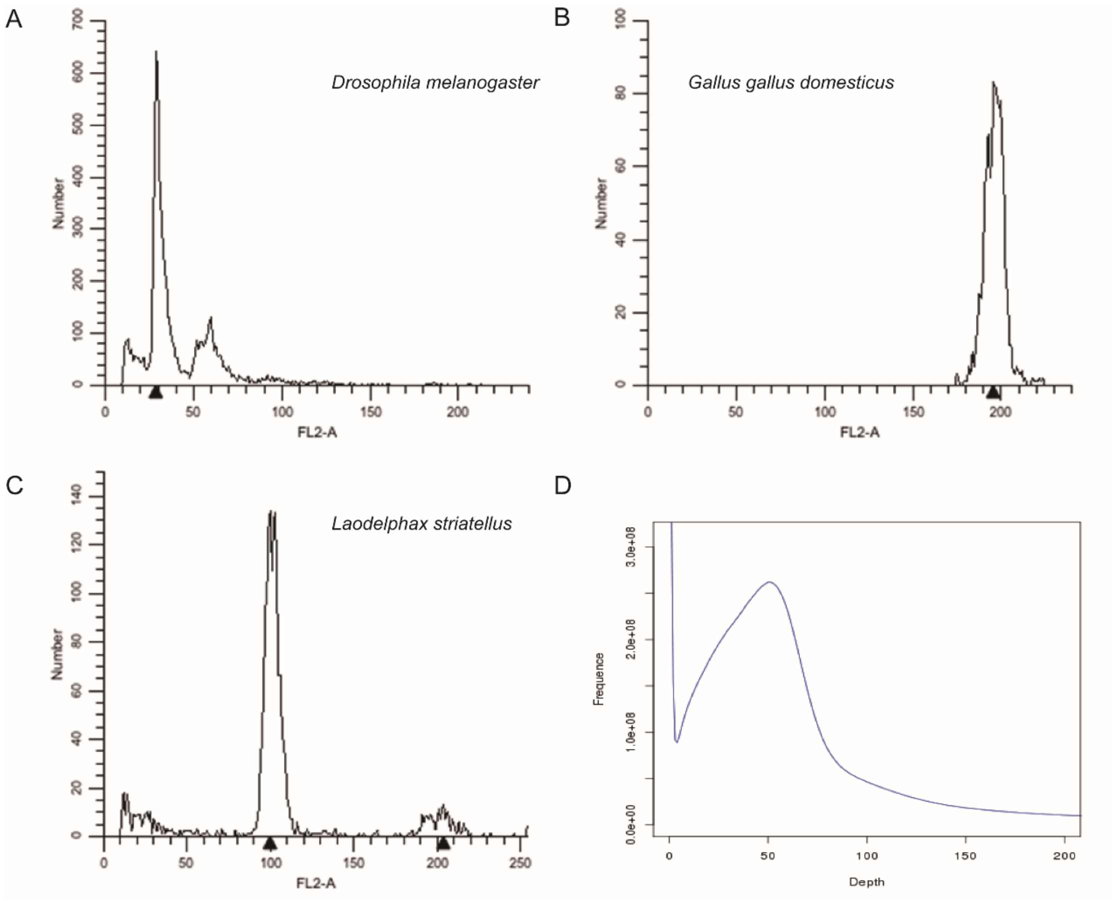

Figure S1. *Laodelphax striatellus* genome size estimation by flow cytometry and k-mer analyses. (A), **(B)** and **(C)** showed fluorescence peaks for *Drosophila melanogaster*, *Gallus gallus* and *L. striatellus*, respectively. The genome sizes of *D. melanogaster* and *G. gallus* were 0.18 pg and 1.25 pg, respectively. The genome size of *L. striatellus* was calculated to be 0.60 pg. **(D)** illustrated the depth distribution of k-mers (k = 17).

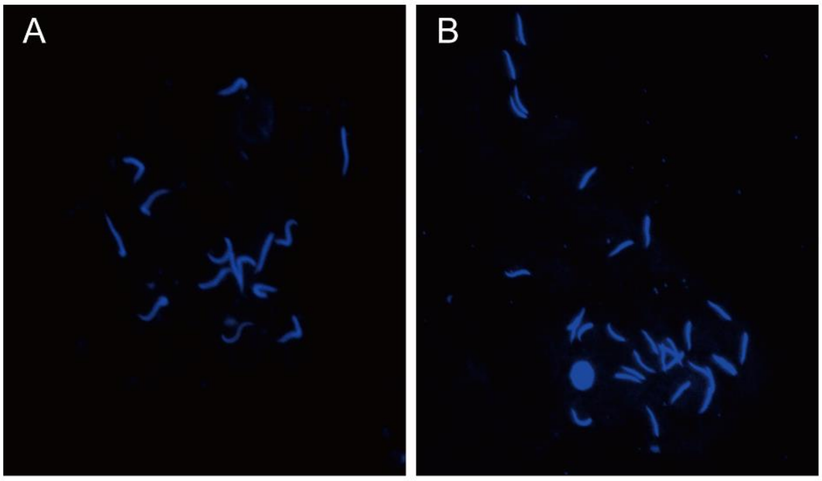

Figure S2. ***Laodelphax striatellus*** **chromosomes dyed with Hoechst 33258. (A)** haploid chromosomes. (**B)** diploid chromosomes.

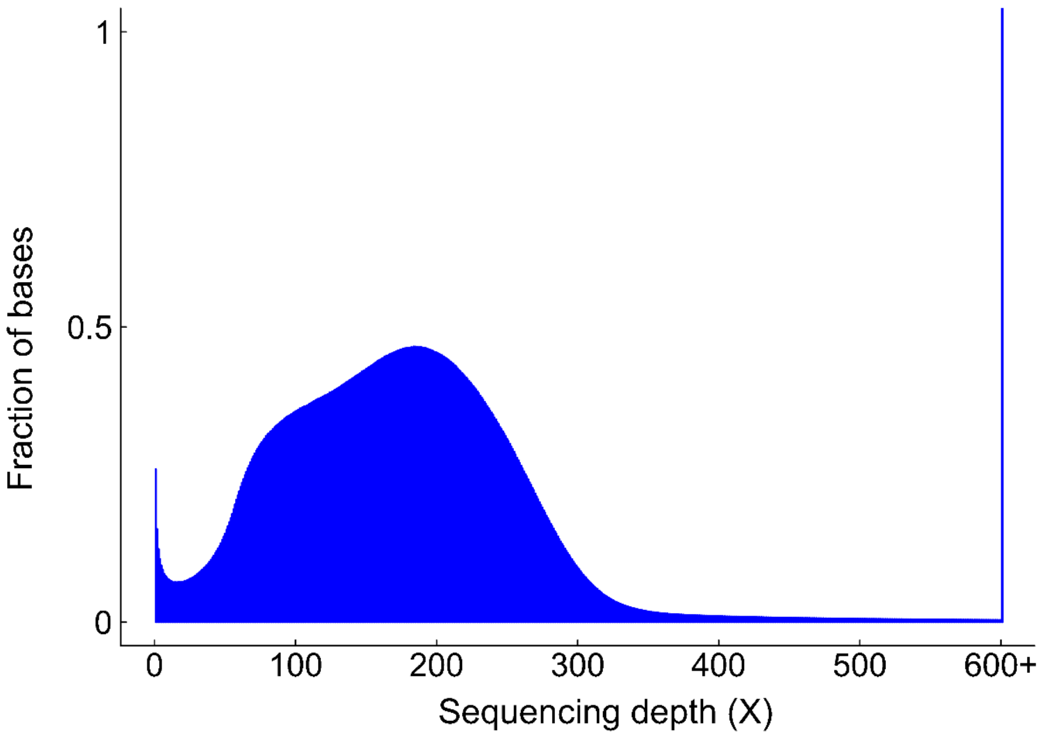

Figure S3. Sequencing depth distribution. The x-axis shows sequencing depth and the y-axis shows fraction of bases with certain sequencing depth.

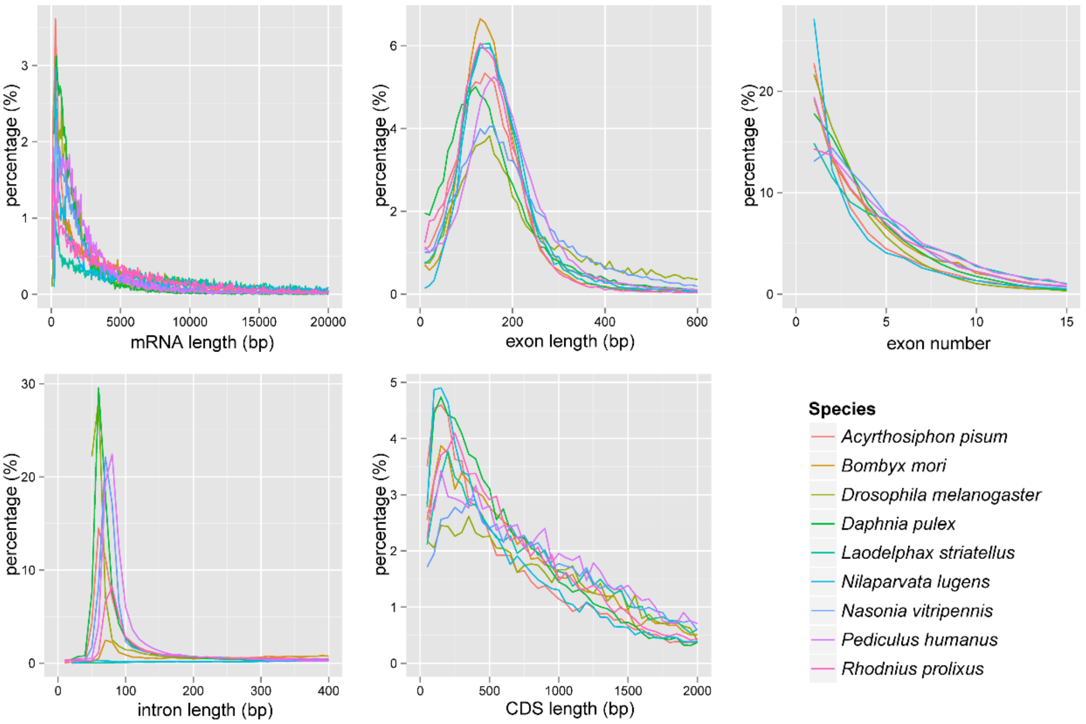

Figure S4. Summary of gene structures of *Laodelphax striatellus* and eight other species used for gene annotation.

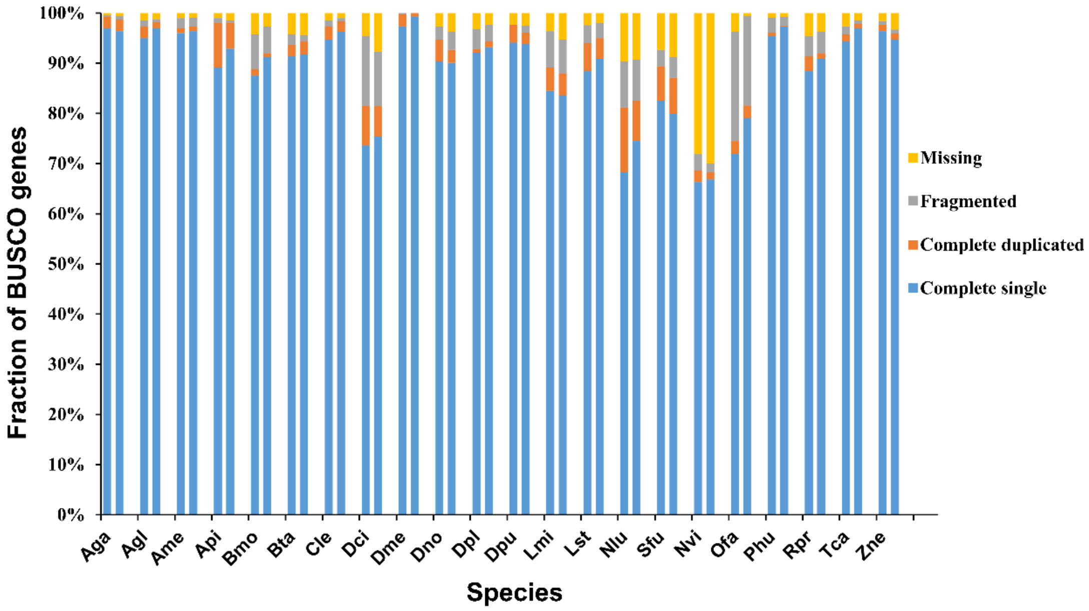

Figure S5. Benchmarking universal single copy orthologs (BUSCO) assessment of the *Laodelphax striatellus* gene set. The completeness of the gene set was assessed with two BUSCO Ver. 2 datasets (arthropoda and eukaryote). The recovered matches are classified as ‘complete’ if their lengths are within the expectation of the BUSCO profile match lengths. If these are found only once they are classified as ‘complete single’ and other ‘complete’ matches are classified as ‘complete duplicated’. The matches that are only partially recovered are classified as ‘fragmented’, and BUSCO groups for which there are no matches that pass the tests of orthology are classified as ‘missing’. For each species, the right bar shows the arthropoda results and the left bar shows the eukaryote results. Aga, *Anopheles gambiae*; Agl, *Anoplophora glabripennis*; Ame, *Apis mellifera*; Api, *Acyrthosiphon pisum*; Bmo, *Bombyx mori*; Bta, *Bemisia tabaci*; Cle, *Cimex lectularius*; Dci, *Diaphorina citri*; Dme, *Drosophila melanogaster*; Dno, *Diuraphis noxia*; Dpl, *Danaus plexippus*; Dpu, *Daphnia pulex*; Lmi, *Locusta migratoria*; Lst, *Laodelphax striatellus*; Nlu, *Nilaparvata lugens*; Nvi, *Nasonia vitripennis*; Ofa, *Oncopeltus fasciatus*; Phu, *Pediculus humanus*; Rpr, *Rhodnius prolixus*; Sfu, *Sogatella furcifera*; Tca, *Tribolium castaneum*; Zne, *Zootermopsis nevadensis*.

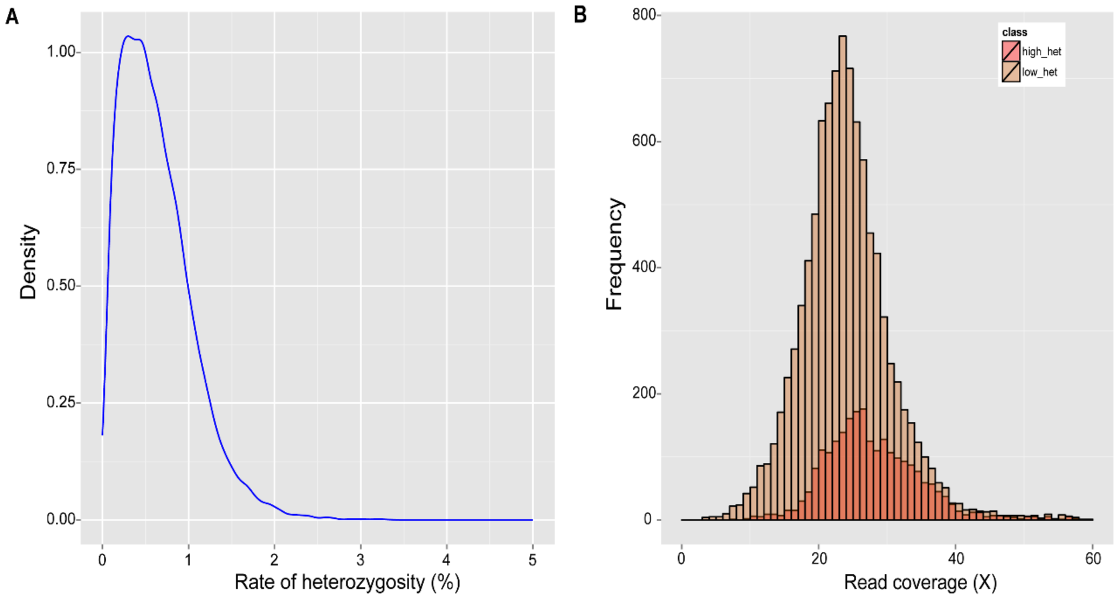

Figure S6. Determination of genomic heterozygosity. (A) Density distribution of heterozygous rates. **(B)** Frequency distribution of read coverage of both high and low heterozygosity. All heterozygosity rates were ranked and the top 20% was chosen as high heterozygosity (high_het in the legend) and the left as low heterozygosity (low_het in the legend).

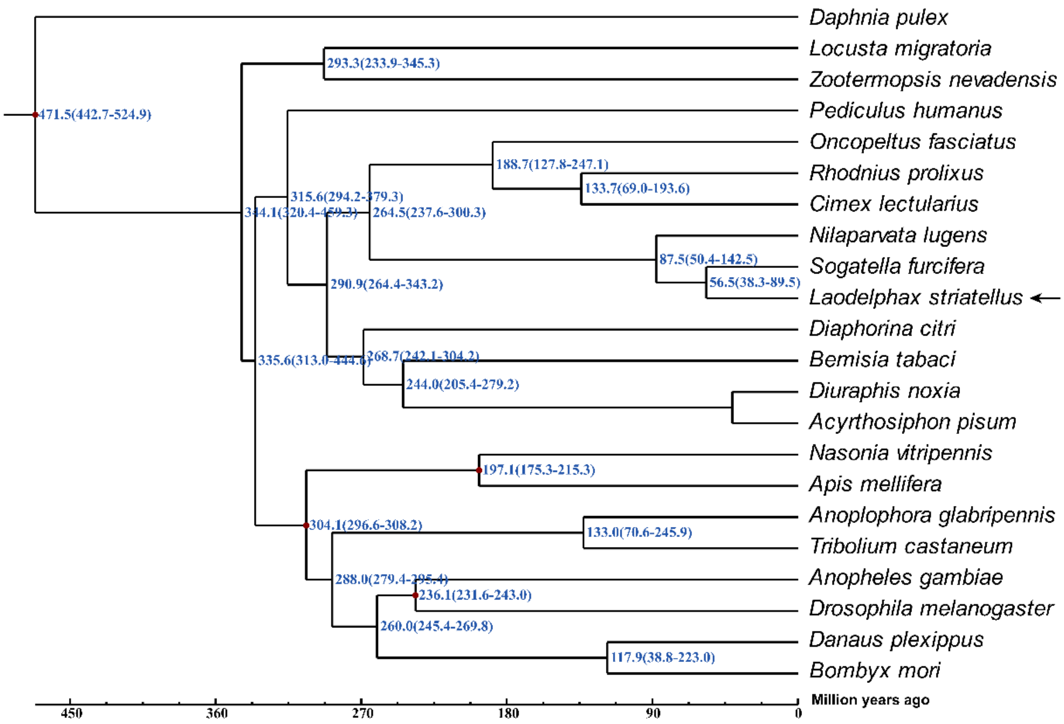

Figure S7. Divergence time estimation of 22 arthropod species. The number on each node stands for the divergence time from the present (million years ago, Mya) with 95% confidence interval values noted in brackets. Four calibration time were used in the estimation: *D. pulex*-*D. melanogaster* divergence (445~530 Mya), *N. vitripennis*-*D. melanogaster* divergence (279~306 Mya), *A. gambiae*-*D. melanogaster* divergence (235~269 Mya) and *A. mellifera*-*N. vitripennis* divergence (175~215 Mya). The location of *L. striatellus* was indicated by an arrow.

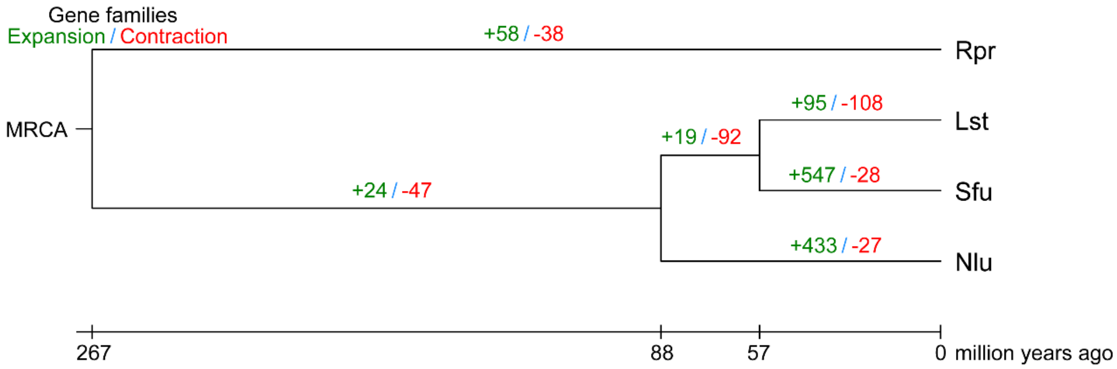

Figure S8. Gene family expansion and contraction in the three planthoppers. *R. prolixus* was used as outgroup to construct the phylogenetic tree and infer expanded/contracted gene families by CAFÉ. A conditional *P*-value was calculated for each gene family and families with P-value < 0.05 were considered as significantly expanded (green) or contracted (red).
