# Supplementary material for: Genome sequence of the small brown planthopper, Laodelphax striatellus
Source: Gigascience. 2017 Nov 10;6(12):1–12. doi: 10.1093/gigascience/gix109 (PMC5740986; doi:10.1093/gigascience/gix109)
Supplement: Response-to-Reviewer-Comments_Original-Submission.pdf [file gix109_response-to-reviewer-comments_original-submission.pdf]

Author's Response To Reviewer Comments

Editor suggestions:
I agree with reviewer 1 that all genome resources, such as contigs, scaffolds, annotated scaffolds should be made easily accessible, for example via the i5k repository mentioned by the reviewer, and via our repository GigaDB.

Response: We contacted i5k and all genome resources are being submitted to the i5k repository. We has uploaded these data to the repository GigaDB (ftp://user9@penguin.genomics.cn).

In your revised manuscript, please include a citation to your GigaDB dataset to your reference list, and cite this in the data availability section and elsewhere in the manuscript, where appropriate.

Response: We included a citation [57] to our GigaDB dataset to the reference list and cited this in the data availability section, Line 450.

Reviewer #1:
The paper is very straightforward, easy to read and the data are strong and very clear. This is a short interesting and comprehensive genome paper with appropriate strategies. I did not see either in the manuscript or in the GigaD repository the access to the genome resources: contigs, scaffolds, annotated scaffolds, browser… Please make it easily accessible. Access to the assembled and annotated genome could be easier into a dedicated database, such the one developed by i5k. I encourage the authors to contact the curators of this databac

Response: We contacted i5k and all genome resources are being submitted to the i5k repository. We has uploaded these data to the repository GigaDB (ftp://user9@penguin.genomics.cn).

It is probable that L. striatellus has bacterial symbiont. Did the authors find some traces of bacterial sequences that could correspond to the symbiont(s)?

Response: We aligned Wolbachia genome to the genome of L. striatellus and found that 96% of Wolbachia genome was covered in the genome of L. striatellus.

I also highlight that the authors made some quantitative and qualitative comparisons with the 2 other planthopper genomes. More particularly, they performed transcriptomic analyses between these three species in terms of virus charge. I suggest the authors to indicate whether the three viruses used for this study have or not the same kind of biology within their host insect: do they replicate for instance in the planthopper host? As well, by searching for GO terms specific to the different conditions, it is not clear whether the authors have used statistical based analysis to see if the enrichment is significant.

Response: Yes, the three viruses used for this study are transmitted in a persistent-

propagative way. We complemented this information in Line 384. We retrieved the common GO terms from respective GO terms specific to different conditions (Lines 405-406), not using the enrichment analysis.

Table 1: please indicate more clearly which library is DNA or RNA.

Response: We added the information of libraries in Table 1 as suggested by the reviewer.

Fig 2 and FigS7: mark with an arrow the location of L. sriatellus

Response: We added an arrow in the location of L. striatellus in these two figures.

Fig 4: I think information is missing such as the divergence between the branches, and the signification of the red spots.

Response: The divergence time between branches were marked in Figure S7. The red spots in Figure 4 were used to show clearly the divergence positions between the branches.

Fig S4: it is difficult to follow with all the different curves of different colors. Is it necessary to show the data for all the species? Would it be better to select some species?

Response: It is not good to use less species in Fig S4. Cross-species density plots using a broad range of insect species can provide a global comparison of gene structure parameter. The comparison results indicate the gene structure parameters of the small brown planthopper don't show an obvious deviation from those of other insects.

Reviewer #2:
The one suggestion I would make is that the observations on host range do not always agree with what was found in the comparison of the Pea aphid and the Soybean aphid. There, the smaller genome was associated with a reduced host range. It is important to make the comparison between the results for the aphid and plant hopper genomes to show that generalizations based only on one or the other will be misleading.

Response: We made the comparison between the results for the aphid and planthpooer genomes in Lines 438-443: Despite having the smallest genome, L. striatellus has the widest host plant range among the three planthoppers. This situation is different from that of the genome evolution in Aphididae, where the soybean aphid, Aphis glycines, which is an extreme specialist, has the smallest genome compared to another three aphid species with published genome sequences [56].

Reference 8 is often cited rather than the original source. Unless the number of references is limited the original source should be cited.

Response: We replaced reference 8 with the original source, the new reference [10], for the flow cytometry in Line 121 and 122.

Keywords should not include words in the title.

Response: We adjusted the keywords as “Comparative genomics; Insects; Genome sequencing; Annotation; Virus transmission”.

Line 129 In total, we. Not "Totally WE"

Response: We made the correction as the reviewer suggested.

Look at S1 Line 157. Solution, fixed in Not, "solution. After fixed" . The later could read as "After fixing" but the latter will be viewed as jargon.

Response: We made the correction as the reviewer suggested.

Comments regarding base composition are either unclear or vague. Line 201 refers to "proper base composition". The term proper is subjective. It is not clear what it means in this context. The same can be said for Line 202. It is not clear what is meant by "The GC content of L. striatellus was 34.54%, similar to that of N. lugens". Is this in reference to overall GC, or based on bin composition? The reader needs more information here. What exactly was done?

Response: We revised these sentences as follows: First, the overall base composition and the percentage of Ns were calculated. As shown in Table S1, the assembled genome had a low percentage (1.99%) of Ns and an expected base composition, which is similar to that of the other two planthoppers. The overall GC content of L. striatellus was 34.54%, similar to that of N. lugens [8] and slightly higher than that of S. furcifera [9].

Line 256 delete "was used".

Response: "was used" was deleted.

Line 421. Missing word, "We referred to genes"

Response: “to” was added here.
