# Supplementary material for: Genome sequence of the small brown planthopper, Laodelphax striatellus
Source: Gigascience. 2017 Nov 10;6(12):1–12. doi: 10.1093/gigascience/gix109 (PMC5740986; doi:10.1093/gigascience/gix109)
Supplement: Reviewer-1-Report-(Original-Submission) -- Denis Tagu — 23 Aug 2017 Reviewed [file gix109_reviewer-1-report-(original-submission).pdf]

Reviewer Report

Title: Genome sequence of the small brown planthopper Laodelphax striatellus

Version: Original Submission **Date:** 23 Aug 2017

Reviewer name: Denis Tagu

Reviewer Comments to Author:

The manuscript of Zhu et al. deals with the description of the genome of the planthopper Laodelphax striatellus. This is the third planthopper genome to be sequenced, and the authors take this opportunity to make some comparisons between those three plant pests that have some different biologies, such as the host-range and the types of viruses they transmit.

The paper is very straightforward, easy to read and the data are strong and very clear. This is a short interesting and comprehensive genome paper with appropriate strategies.
I did not see either in the manuscript or in the GigaD repository the access to the genome resources: contigs, scaffolds, annotated scaffolds, browser… Please make it easily accessible. Access to the assembled and annotated genome could be easier into a dedicated database, such the one developed by i5k. I encourage the authors to contact the curators of this database that could home the L. striatellus genome: https://i5k.nal.usda.gov/
I have no major recommendations since all the steps for acquiring and analyzing the genome were correctly performed, such as sequencing strategy (Illumina + PacBio), sequence coverage, assembly that provided a good quality for a draft genome. May be the authors could indicate how many or proportion of "short" scaffolds they have, as well as (or) giving the number of scaffolds that cover ½ of the assembled genome. This will help to see whether the still large number of scaffolds is due to small ones.
I appreciated the annotations of TE and protein-coding genes with different QCs to demonstrate that most (if not all) of the genes have been predicted.
It is probable that L. striatellus has bacterial symbiont. Did the authors find some traces of bacterial sequences that could correspond to the symbiont(s)?
I also highlight that the authors made some quantitative and qualitative comparisons with the 2 other planthopper genomes. More particularly, they performed transcriptomic analyses between these three species in terms of virus charge. I suggest the authors to indicate whether the three viruses used for this study have or not the same kind of biology within their host insect: do they replicate for instance in the planthopper host? As well, by searching for GO terms specific to the different conditions, it is not clear whether the authors have used statistical based analysis to see if the enrichment is significant.
Table 1: please indicate more clearly which library is DNA or RNA.
Fig 2 and FigS7: mark with an arrow the location of L. sriatellus
Fig 4: I think information is missing such as the divergence between the branches, and the signification of the red spots.
Fig S4: it is difficult to follow with all the different curves of different colors. Is it necessary to show the data for all the species? Would it be better to select some species?

Level of Interest

Please indicate how interesting you found the manuscript: An article whose findings are important to those with closely related research interests

Quality of Written English

Please indicate the quality of language in the manuscript: Needs some language corrections before being published

Declaration of Competing Interests

Please complete a declaration of competing interests, considering the following questions:

- Have you in the past five years received reimbursements, fees, funding, or salary from an organisation that may in any way gain or lose financially from the publication of this manuscript, either now or in the future?
- Do you hold any stocks or shares in an organisation that may in any way gain or lose financially from the publication of this manuscript, either now or in the future?
- Do you hold or are you currently applying for any patents relating to the content of the manuscript?
- Have you received reimbursements, fees, funding, or salary from an organization that holds or has applied for patents relating to the content of the manuscript?
- Do you have any other financial competing interests?
- Do you have any non-financial competing interests in relation to this paper?

If you can answer no to all of the above, write 'I declare that I have no competing interests' below. If your reply is yes to any, please give details below.

I declare that I have no competing interests

I agree to the open peer review policy of the journal. I understand that my name will be included on my report to the authors and, if the manuscript is accepted for publication, my named report including any attachments I upload will be posted on the website along with the authors' responses. I agree for my report to be made available under an Open Access Creative Commons CC-BY license (http://creativecommons.org/licenses/by/4.0/). I understand that any comments which I do not wish to be included in my named report can be included as confidential comments to the editors, which will not be published.

I agree to the open peer review policy of the journal
