# Supplementary material for: Genome sequence of the small brown planthopper, Laodelphax striatellus
Source: Gigascience. 2017 Nov 10;6(12):1–12. doi: 10.1093/gigascience/gix109 (PMC5740986; doi:10.1093/gigascience/gix109)
Supplement: Reviewer-2-Report-(Original-Submission) -- Spencer Johnston — 19 Sep 2017 Reviewed [file gix109_reviewer-2-report-(original-submission).pdf]

Reviewer Report

Title: Genome sequence of the small brown planthopper Laodelphax striatellus

Version: Original Submission **Date:** 19 Sep 2017

Reviewer name: Spencer Johnston

Reviewer Comments to Author:

The GigaScience paper describes the complete sequence of L. striatellus and compares that to the complete sequences of two other plant hoppers. Evidence is presented to show the sequence is very complete and well assembled. Details of the annotation and assembly process and encorporation of transcriptomic data are clear and complete. A number of interesting comparisons are found that relate to the wider host range of L. striateluus. The number of gene copies of gene families is compared for the three plant hoppers as is the transcriptome of the three when challenged by a virus. With only a few exceptions noted below the paper is well written and clear.
The one suggestions I would make is that the observations on host range do not always agree with what was found in the comparison of the Pea aphid and the Soybean aphid. There, the smaller genome was associated with a reduced host range. It is important to make the comparison between the results for the aphid and plant hopper genomes to show that generalizations based only on one or the other will be misleading.

Reference 8 is often cited rather than the original source. Unless the number of references is limited the original source should be cited.

Keywords should not include words in the title.
Line 129 In total, we. Not "Totally WE"
Look at S1 Line 157. Solution, fixed in Not, "solution. After fixed" . The later could read as "After fixing" but the latter will be viewed as jargon.
Comments regarding base composition are either unclear or vague. Line 201 refers to "proper base composition". The term proper is subjective. It is not clear what it means in this context. The same can be said for Line 202. It is not clear what is meant by "The GC content of L. striatellus was 34.54%, similar to that of N. lugens". Is this in reference to overall GC, or based on bin composition? The reader needs more information here. What exactly was done?
Line 256 delete "was used".
Line 421. Missing word, "We referred to genes"

Level of Interest

Please indicate how interesting you found the manuscript: An exceptional article

Quality of Written English

Please indicate the quality of language in the manuscript: Acceptable

Declaration of Competing Interests

Please complete a declaration of competing interests, considering the following questions:

- Have you in the past five years received reimbursements, fees, funding, or salary from an organisation that may in any way gain or lose financially from the publication of this manuscript, either now or in the future?
- Do you hold any stocks or shares in an organisation that may in any way gain or lose financially from the publication of this manuscript, either now or in the future?
- Do you hold or are you currently applying for any patents relating to the content of the manuscript?
- Have you received reimbursements, fees, funding, or salary from an organization that holds or has applied for patents relating to the content of the manuscript?
- Do you have any other financial competing interests?
- Do you have any non-financial competing interests in relation to this paper?

If you can answer no to all of the above, write 'I declare that I have no competing interests' below. If your reply is yes to any, please give details below.

I declare that I have no competing interests

I agree to the open peer review policy of the journal. I understand that my name will be included on my report to the authors and, if the manuscript is accepted for publication, my named report including any attachments I upload will be posted on the website along with the authors' responses. I agree for my report to be made available under an Open Access Creative Commons CC-BY license (http://creativecommons.org/licenses/by/4.0/). I understand that any comments which I do not wish to be included in my named report can be included as confidential comments to the editors, which will not be published.

I agree to the open peer review policy of the journal
